# Supplementary material for: HtrA3: a promising prognostic biomarker and therapeutic target for head and neck squamous cell carcinoma
Source: PeerJ. 2023 Oct 10;11:e16237. doi: 10.7717/peerj.16237 (PMC10573296; doi:10.7717/peerj.16237)
Supplement: Supplemental Information 1 [file peerj-11-16237-s001.zip › Raw data A/Supplementary Table.docx]

Supplementary Table 1. Primer sequences for qRT-PCR.

| Name | Primer F (sequence 5’-3’) | Primer R (sequence 5’-3’) |
| --- | --- | --- |
| GAPDH | TGACATCAAGAAGGTGGTGAAGCAG | GTGTCGCTGTTGAAGTCAGAGGAG |
| HTRA1 | GTGGCTAGTGGGTCTGGGTTTATTG | TTCGTAAGTGGCACCGTTCTTCAG |
| HTRA2 | GAGACTGCTAAGCGGCGACAC | TGAATCCTCAGCGTTGCGATGTC |
| HTRA3 | ACATTGCCACCATCAAGATCCATCC | ATGCCCGTTGTCACTGTGTTCTG |
| HTRA4 | TTCACGGCAGCAGGCTTGTTC | CCATTCTGGAGCACCACCTCAATC |
