# Supplementary material for: HtrA3: a promising prognostic biomarker and therapeutic target for head and neck squamous cell carcinoma
Source: PeerJ. 2023 Oct 10;11:e16237. doi: 10.7717/peerj.16237 (PMC10573296; doi:10.7717/peerj.16237)
Supplement: Supplemental Information 2 [file peerj-11-16237-s002.zip › Raw data GO KEGG GSEA/Raw data-GSEA.docx]

| ID | setSize | enrichmentScore | NES | pvalue | p.adjust | qvalue |
| --- | --- | --- | --- | --- | --- | --- |
| KEGG_OXIDATIVE_PHOSPHORYLATION | 24 | 0.6949955 | 2.026471 | 0.0001 | 0.0104 | 0.0086 |
| REACTOME_THE_CITRIC_ACID_TCA_CYCLE_AND_RESPIRATORY_ELECTRON_TRANSPORT | 29 | 0.6593472 | 2.004267 | 7.2e-05 | 0.0104 | 0.0086 |
| REACTOME_RESPIRATORY_ELECTRON_TRANSPORT | 28 | 0.6501578 | 1.962995 | 0.0002 | 0.0104 | 0.0086 |
| REACTOME_RESPIRATORY_ELECTRON_TRANSPORT_ATP_SYNTHESIS_BY_CHEMIOSMOTIC_COUPLING_AND_HEAT_PRODUCTION_BY_UNCOUPLING_PROTEINS | 28 | 0.6501578 | 1.962995 | 0.0002 | 0.0104 | 0.0086 |
| KEGG_CARDIAC_MUSCLE_CONTRACTION | 21 | 0.6910468 | 1.922093 | 0.0005 | 0.0251 | 0.0208 |
| WP_HOSTPATHOGEN_INTERACTION_OF_HUMAN_CORONAVIRUSES_APOPTOSIS | 13 | -0.7185709 | -1.972958 | 0.0016 | 0.0348 | 0.0288 |
| WP_FAS_LIGAND_PATHWAY_AND_STRESS_INDUCTION_OF_HEAT_SHOCK_PROTEINS | 17 | -0.6576734 | -1.968384 | 0.0016 | 0.0348 | 0.0288 |
| KEGG_RIG_I_LIKE_RECEPTOR_SIGNALING_PATHWAY | 15 | -0.6774612 | -1.952287 | 0.0016 | 0.0348 | 0.0288 |
| WP_NOVEL_INTRACELLULAR_COMPONENTS_OF_RIGILIKE_RECEPTOR_PATHWAY | 15 | -0.6774612 | -1.952287 | 0.0016 | 0.0348 | 0.0288 |
| KEGG_TOLL_LIKE_RECEPTOR_SIGNALING_PATHWAY | 20 | -0.6209131 | -1.945614 | 0.0019 | 0.0348 | 0.0288 |
