# Supplementary figures and images for: HtrA3: a promising prognostic biomarker and therapeutic target for head and neck squamous cell carcinoma
Source: PeerJ. 2023 Oct 10;11:e16237. doi: 10.7717/peerj.16237 (PMC10573296; doi:10.7717/peerj.16237)

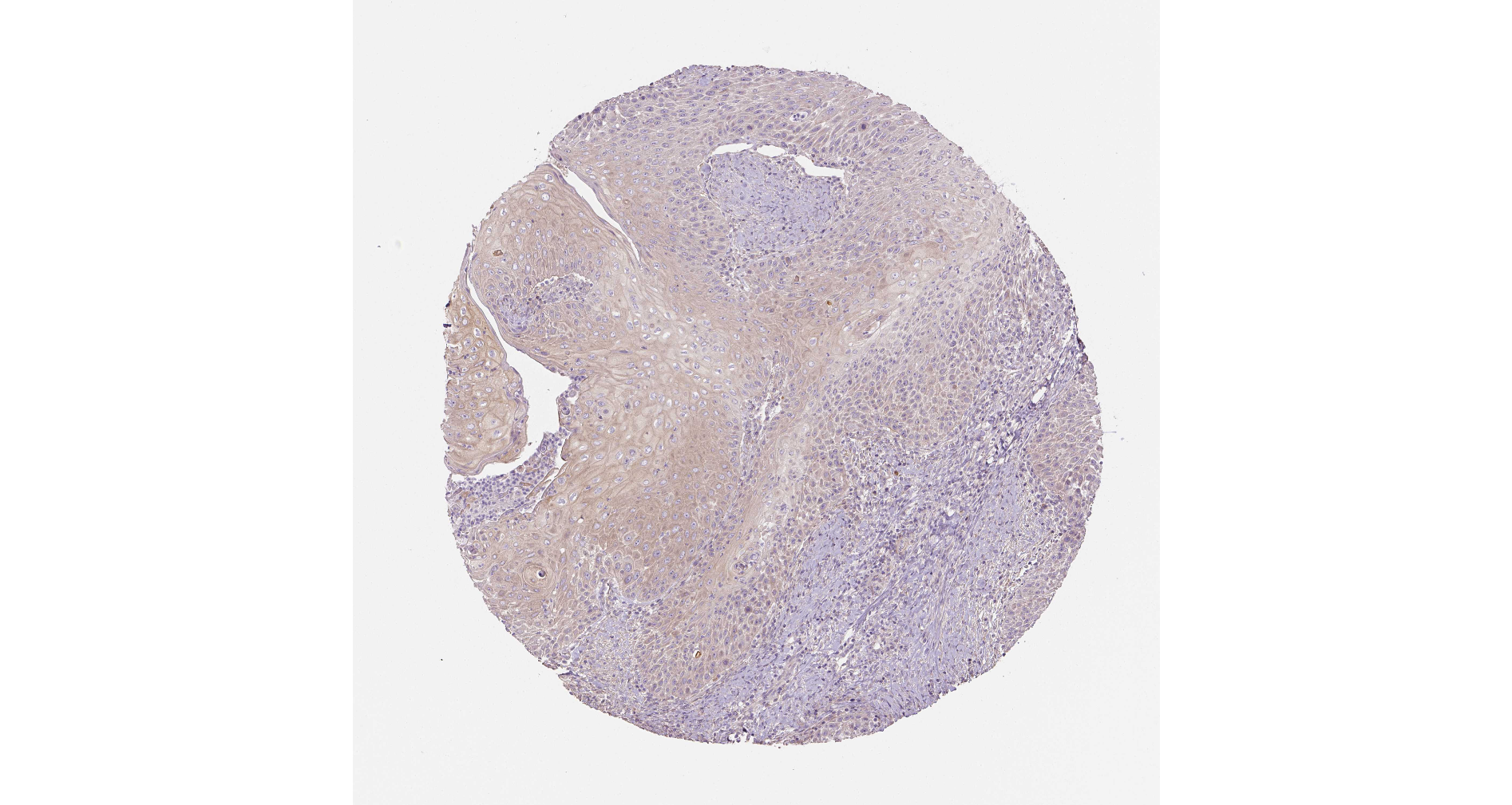

Supplement: Supplemental Information 4 [file peerj-11-16237-s004.zip › Raw data-HPA Validation/HTRA1-HNSCC.png]

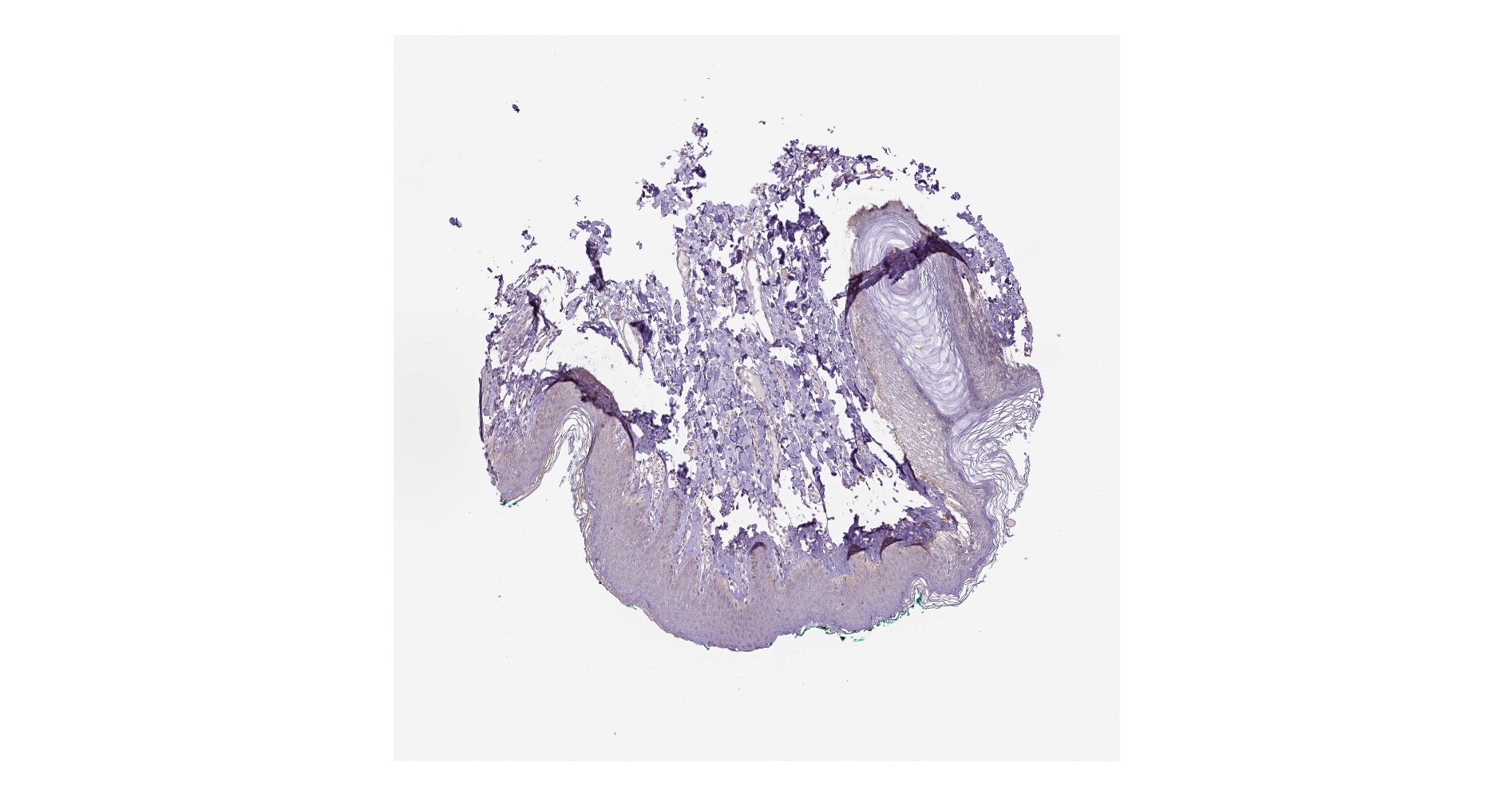

Supplement: Supplemental Information 4 [file peerj-11-16237-s004.zip › Raw data-HPA Validation/HTRA1-Normal.png]

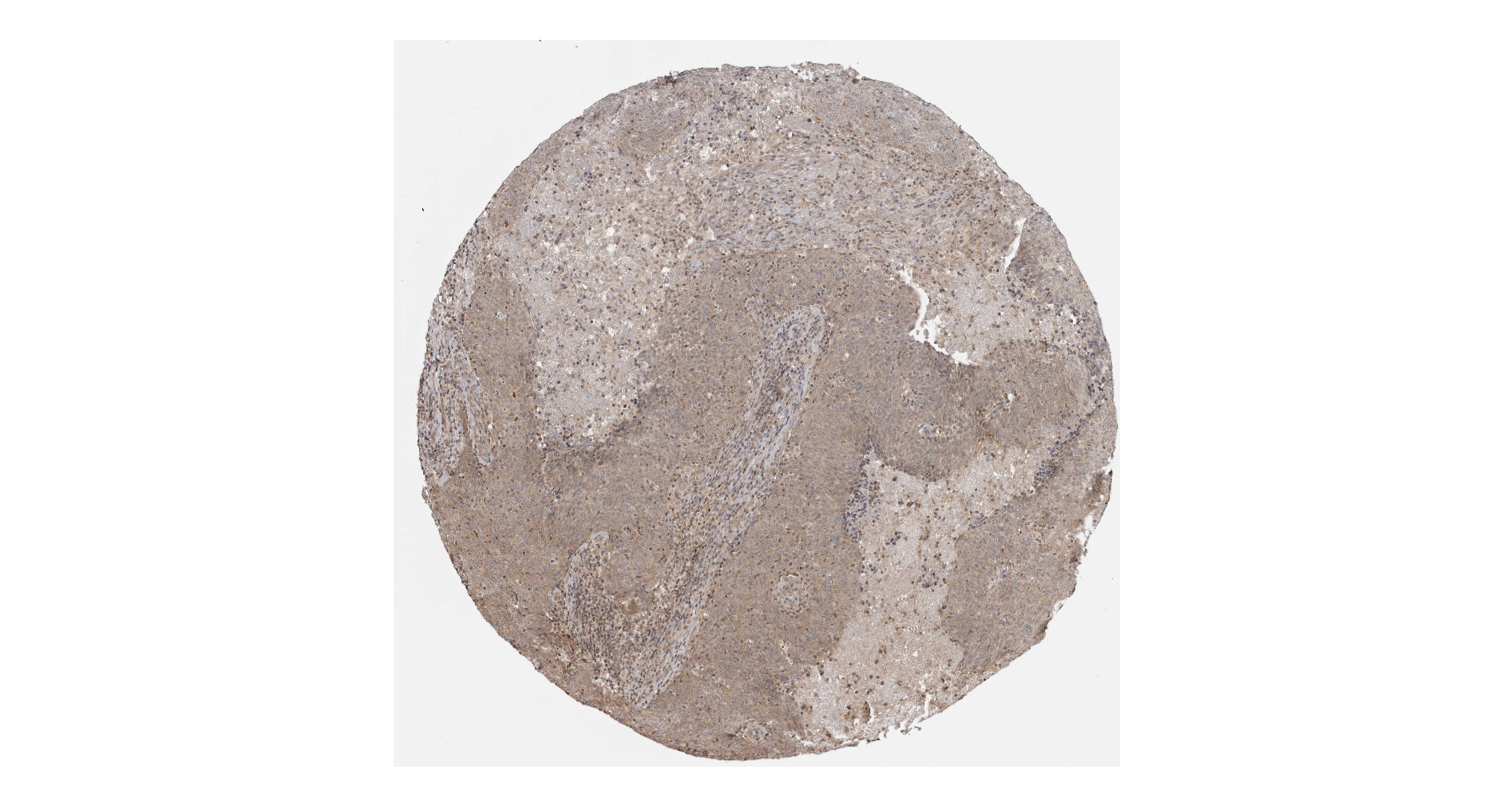

Supplement: Supplemental Information 4 [file peerj-11-16237-s004.zip › Raw data-HPA Validation/HTRA2-HNSCC.png]

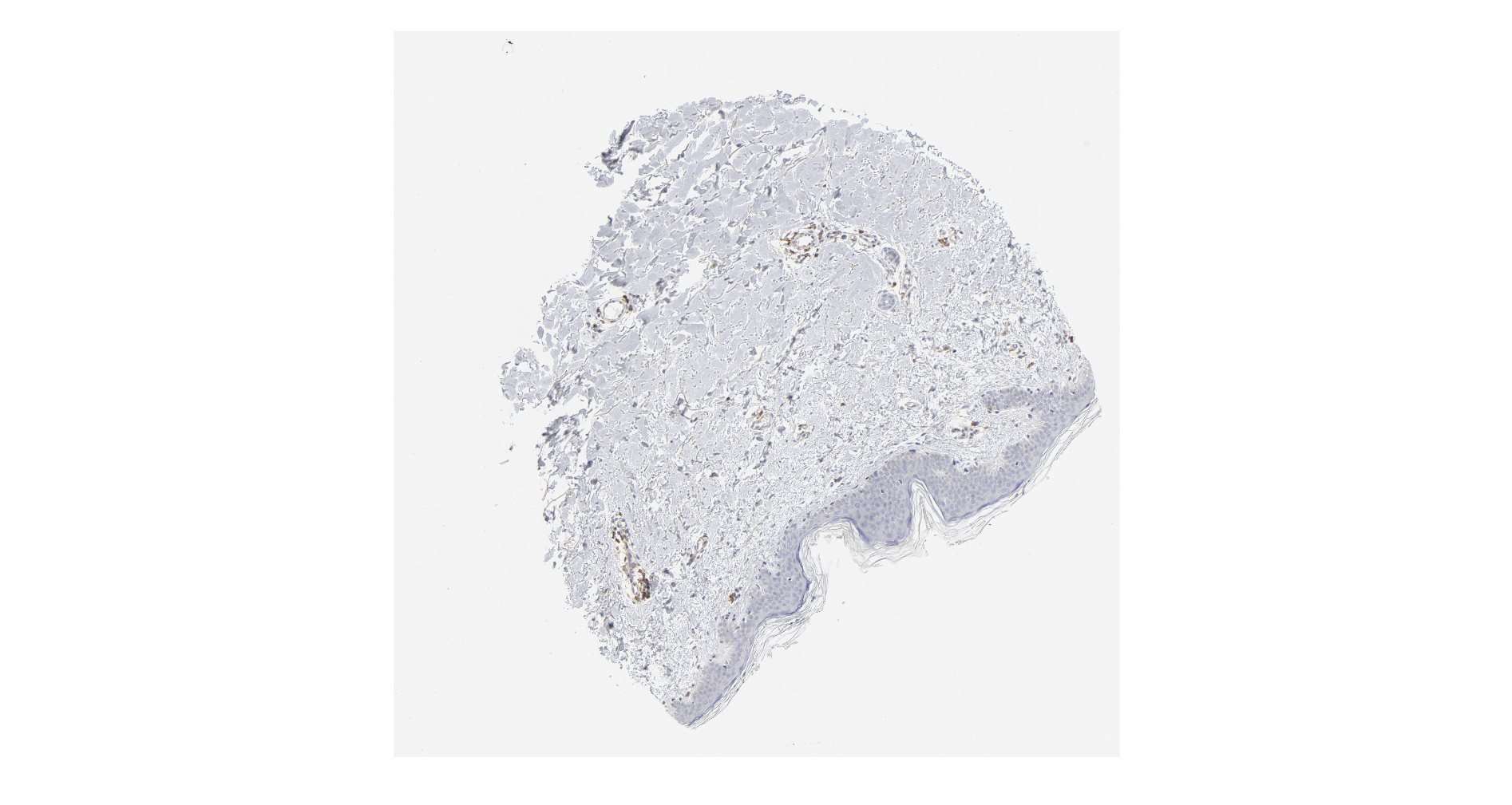

Supplement: Supplemental Information 4 [file peerj-11-16237-s004.zip › Raw data-HPA Validation/HTRA2-Normal.png]

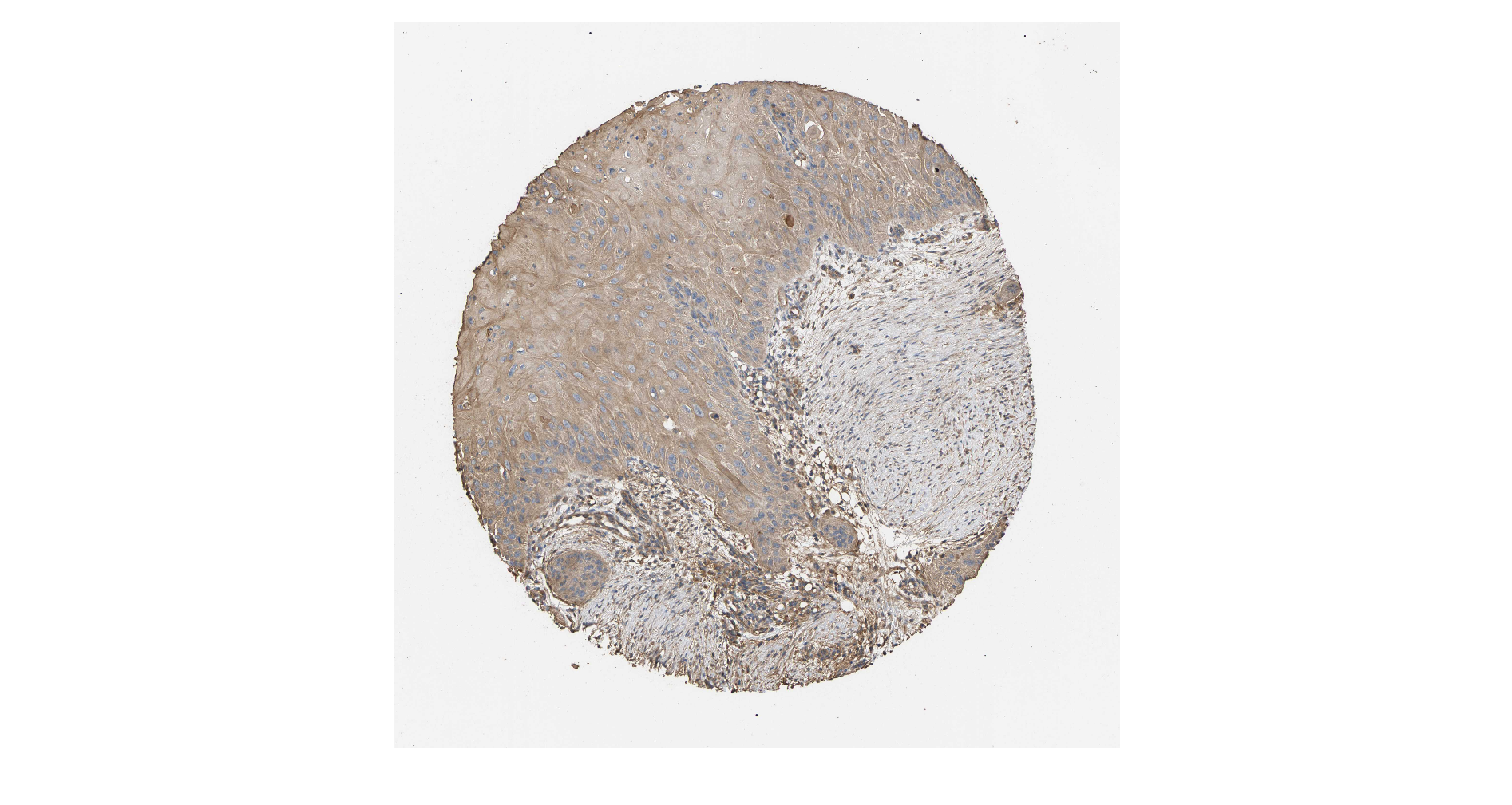

Supplement: Supplemental Information 4 [file peerj-11-16237-s004.zip › Raw data-HPA Validation/HTRA3-HSSCC.png]

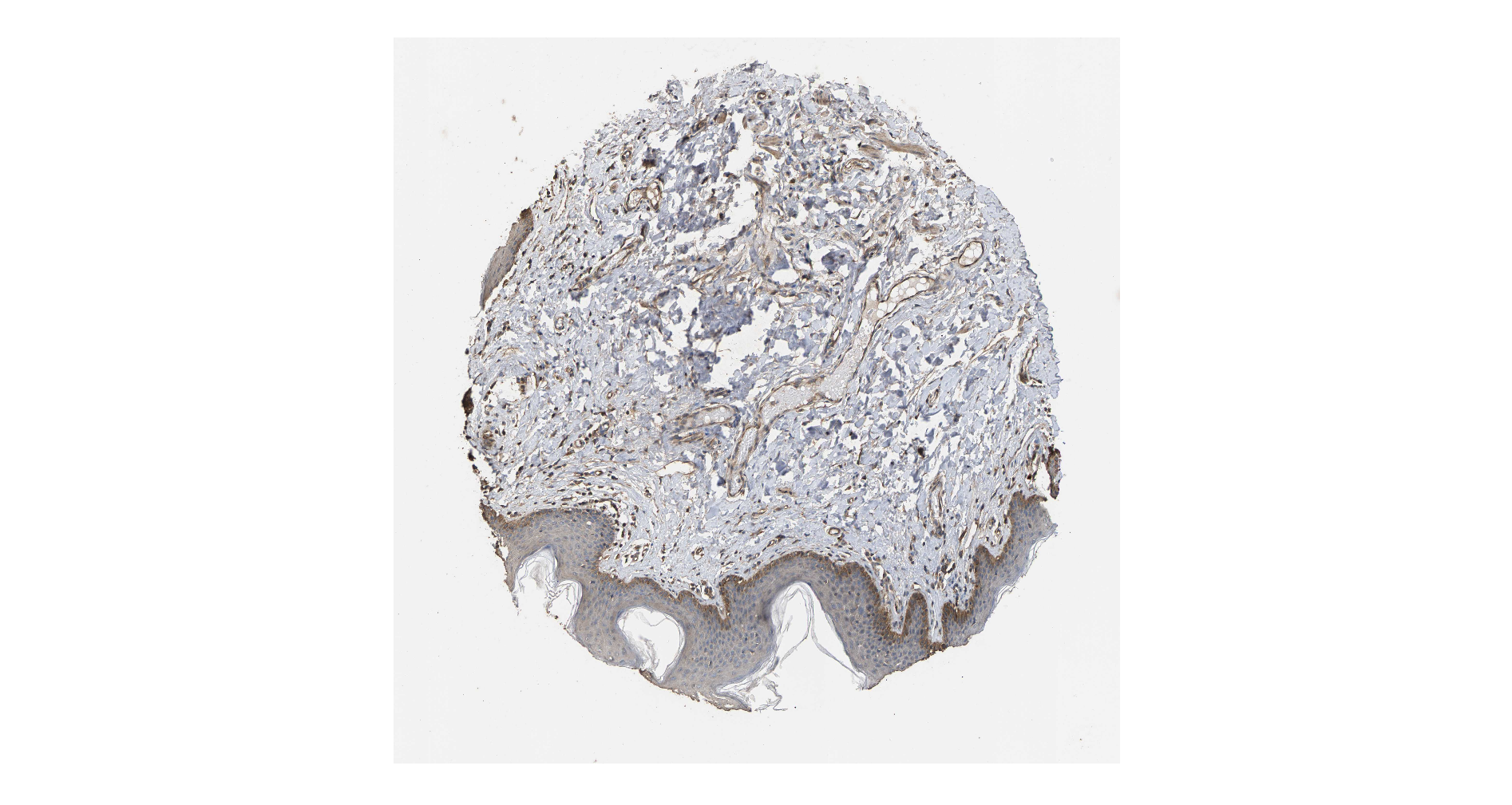

Supplement: Supplemental Information 4 [file peerj-11-16237-s004.zip › Raw data-HPA Validation/HTRA3-Normal.png]

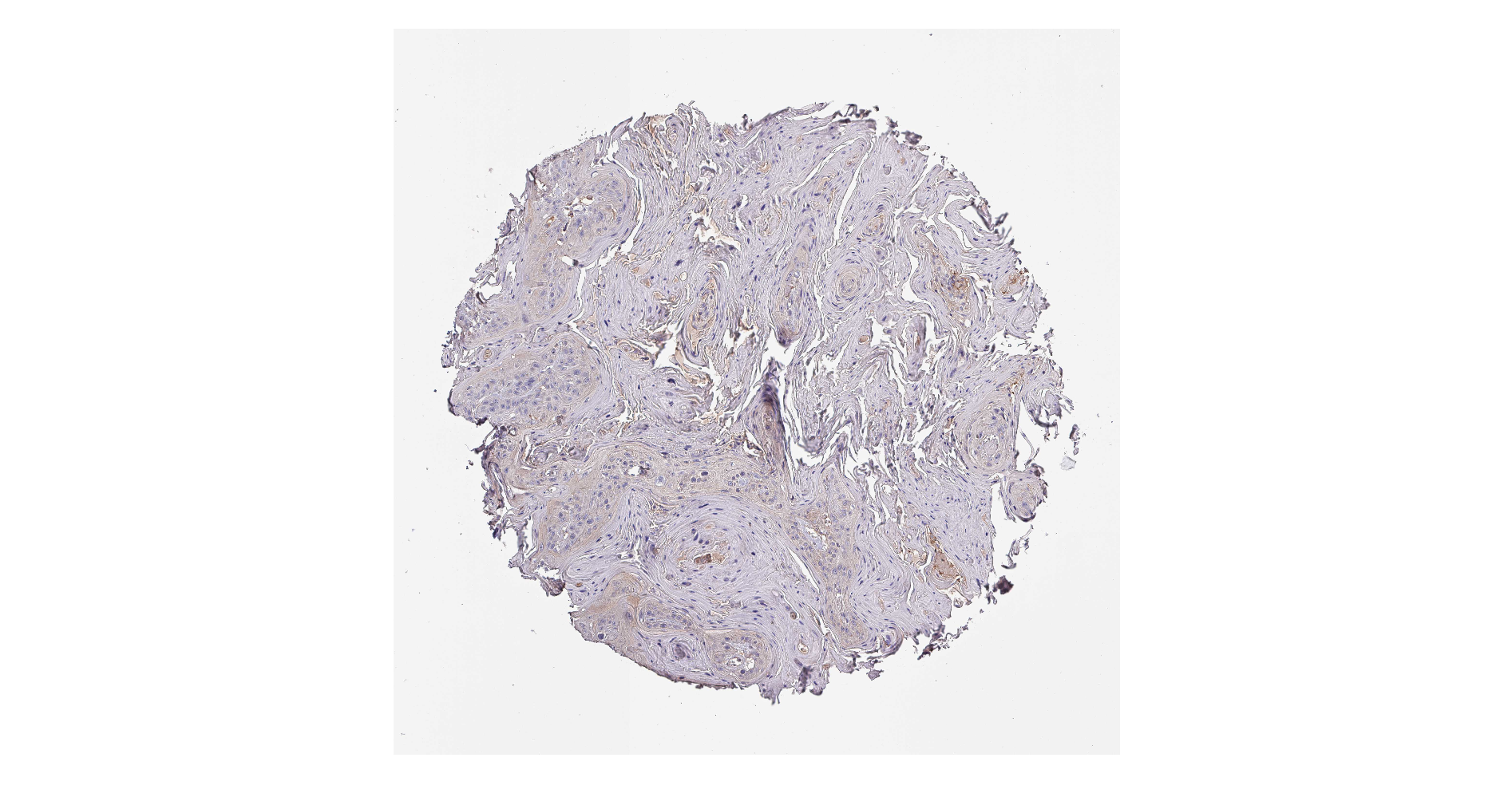

Supplement: Supplemental Information 4 [file peerj-11-16237-s004.zip › Raw data-HPA Validation/HTRA4-HNSCC.png]

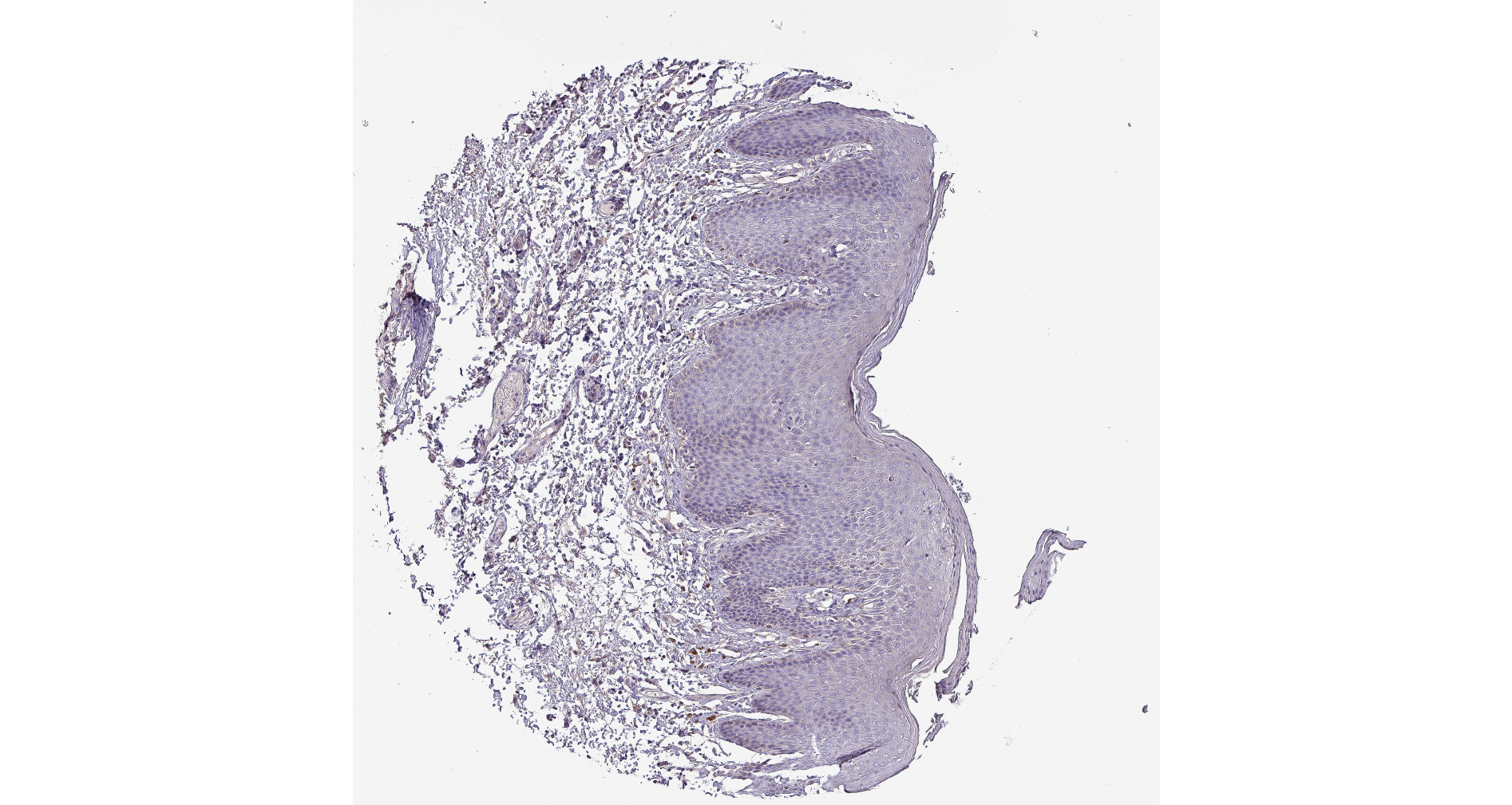

Supplement: Supplemental Information 4 [file peerj-11-16237-s004.zip › Raw data-HPA Validation/HTRA4-Normal.png]

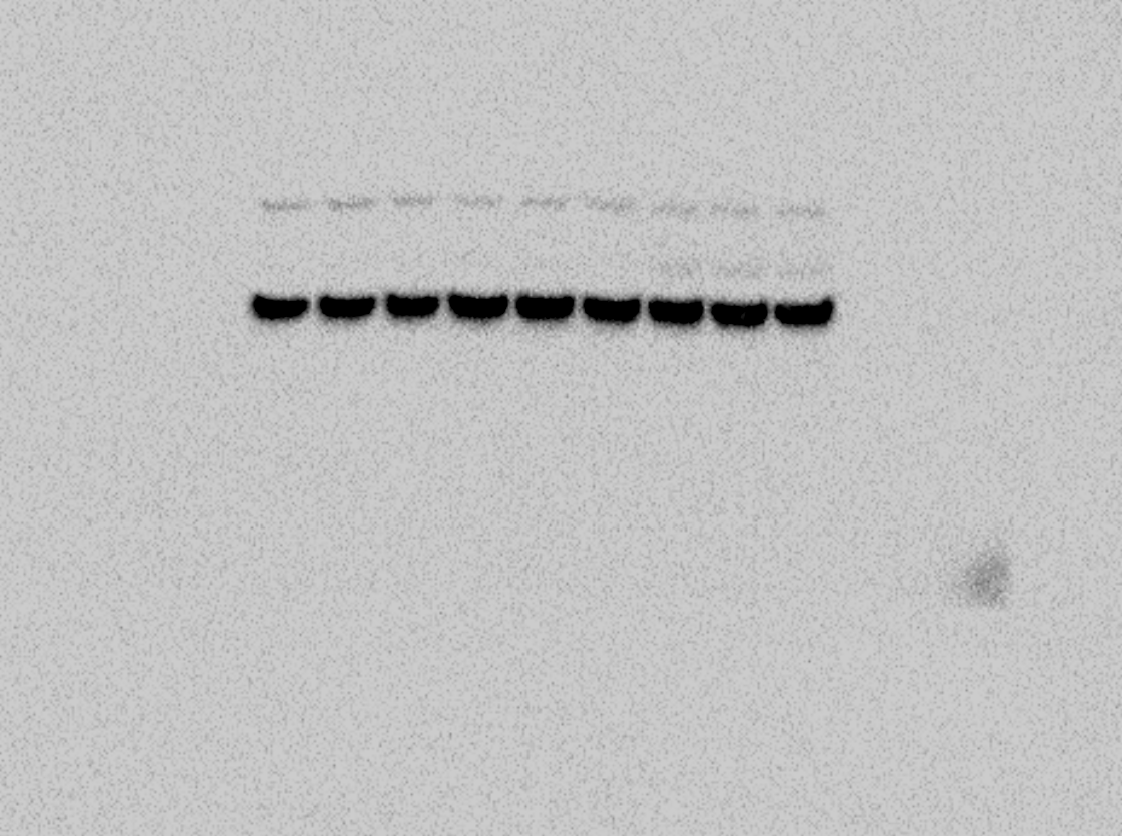

Supplement: Supplemental Information 6 [file peerj-11-16237-s006.zip › Raw data-WB Validation/Cal-27/ACTIN-1.tif]

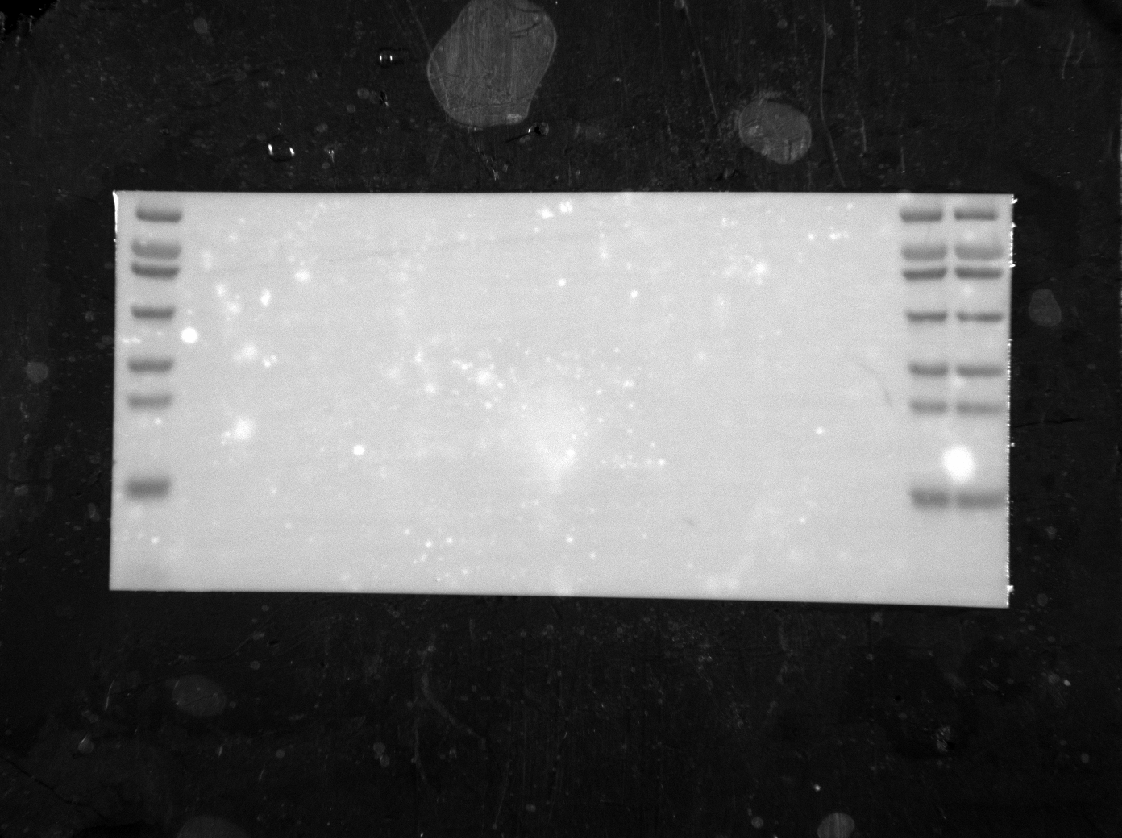

Supplement: Supplemental Information 6 [file peerj-11-16237-s006.zip › Raw data-WB Validation/Cal-27/ACTIN-MARKER.tif]

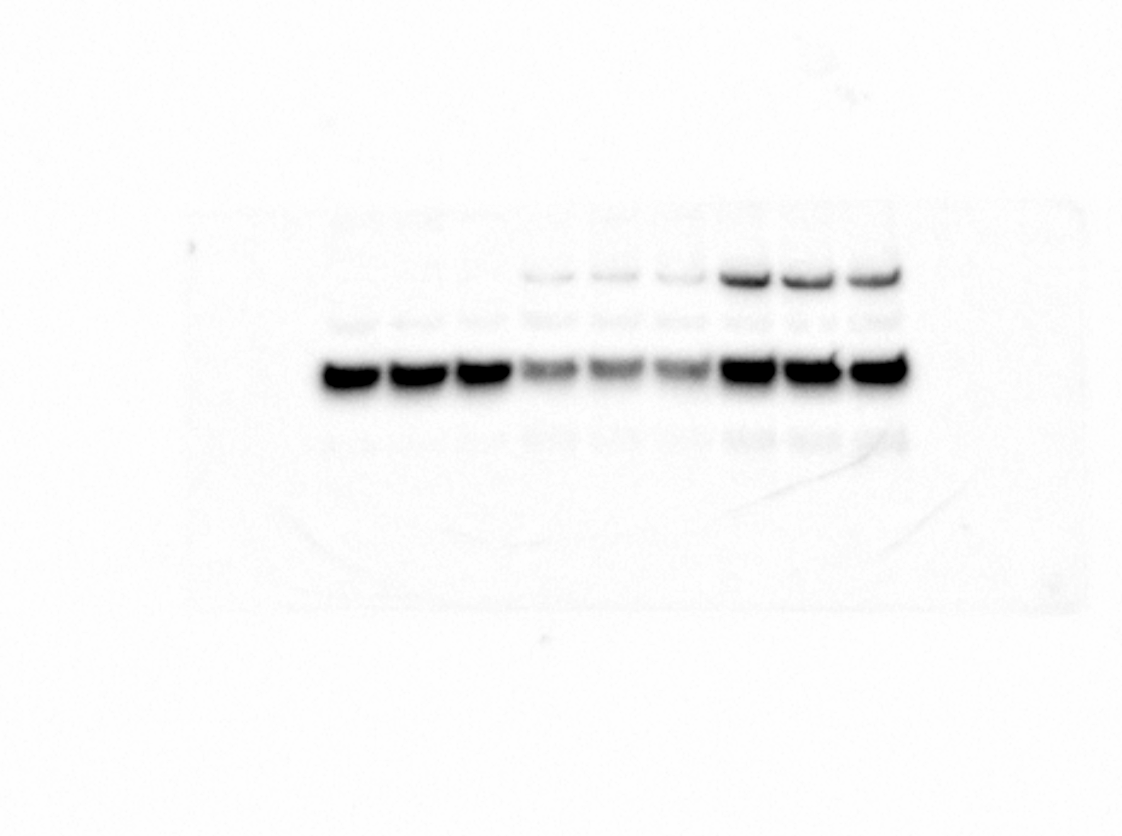

Supplement: Supplemental Information 6 [file peerj-11-16237-s006.zip › Raw data-WB Validation/Cal-27/HTRA1-2.tif]

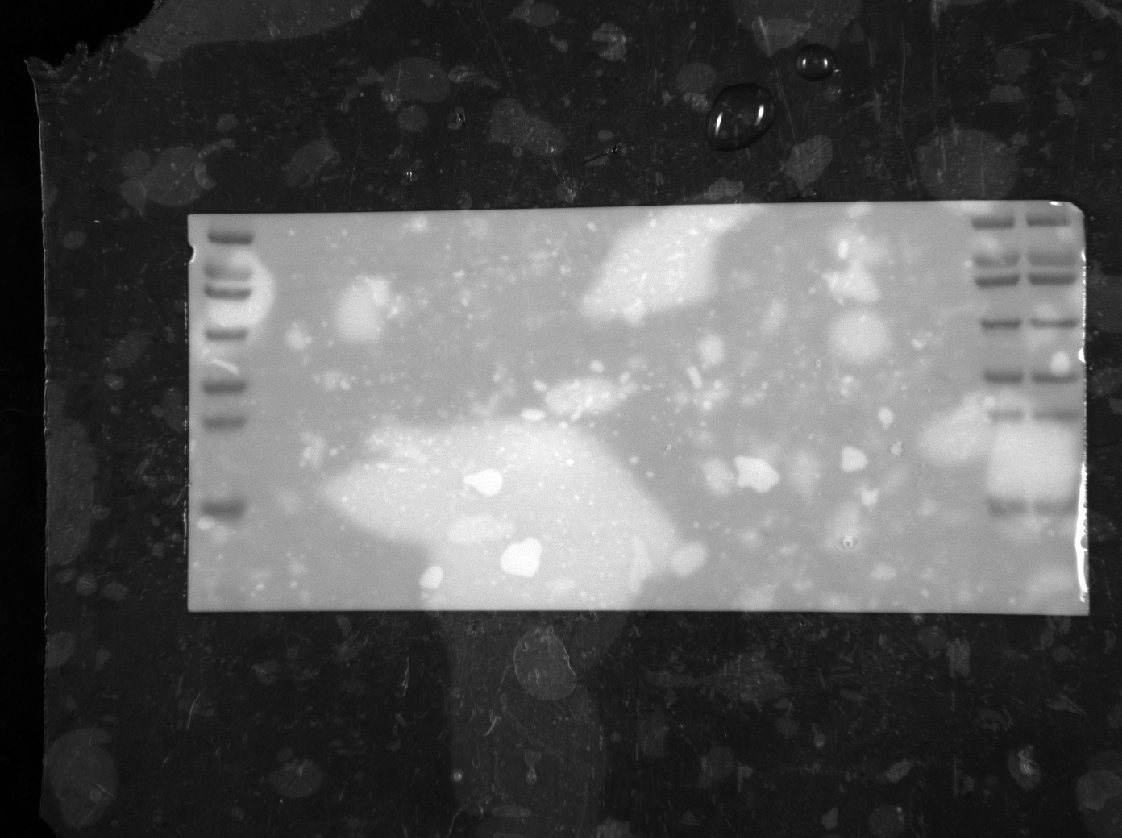

Supplement: Supplemental Information 6 [file peerj-11-16237-s006.zip › Raw data-WB Validation/Cal-27/HTRA1-MARKER.tif]

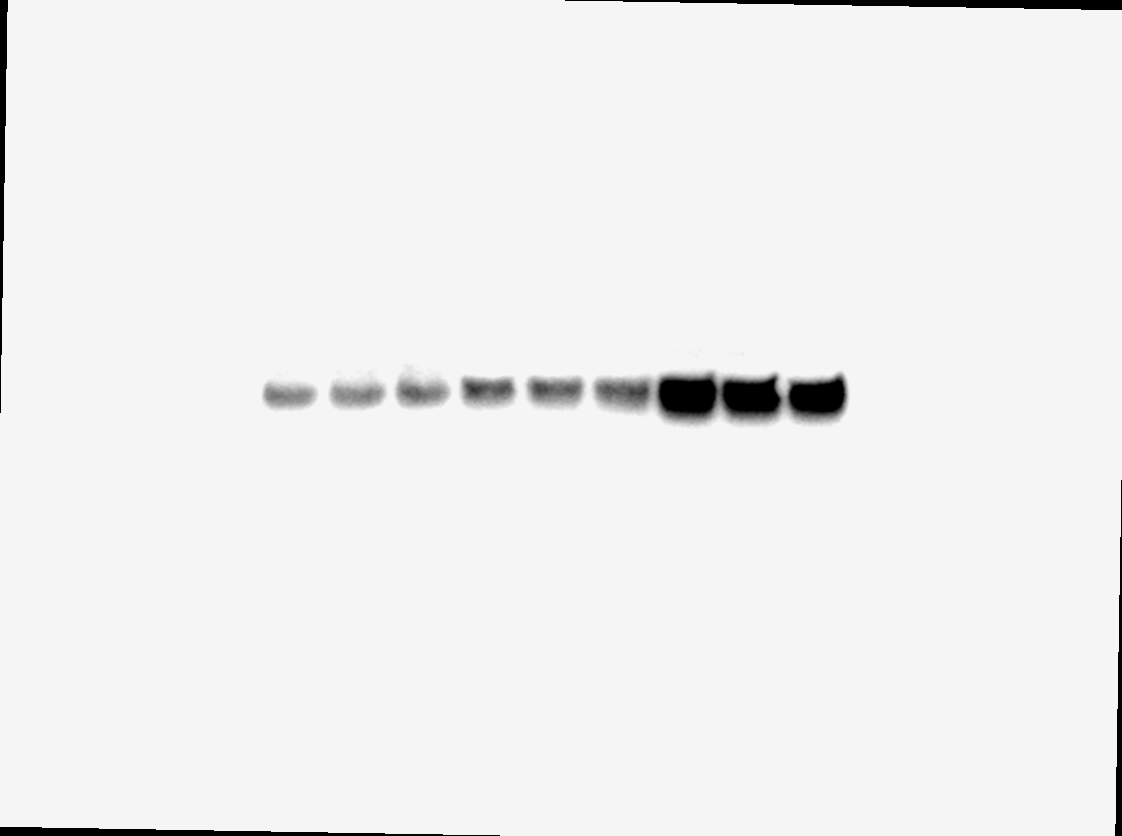

Supplement: Supplemental Information 6 [file peerj-11-16237-s006.zip › Raw data-WB Validation/Cal-27/Htra2-1.tif]

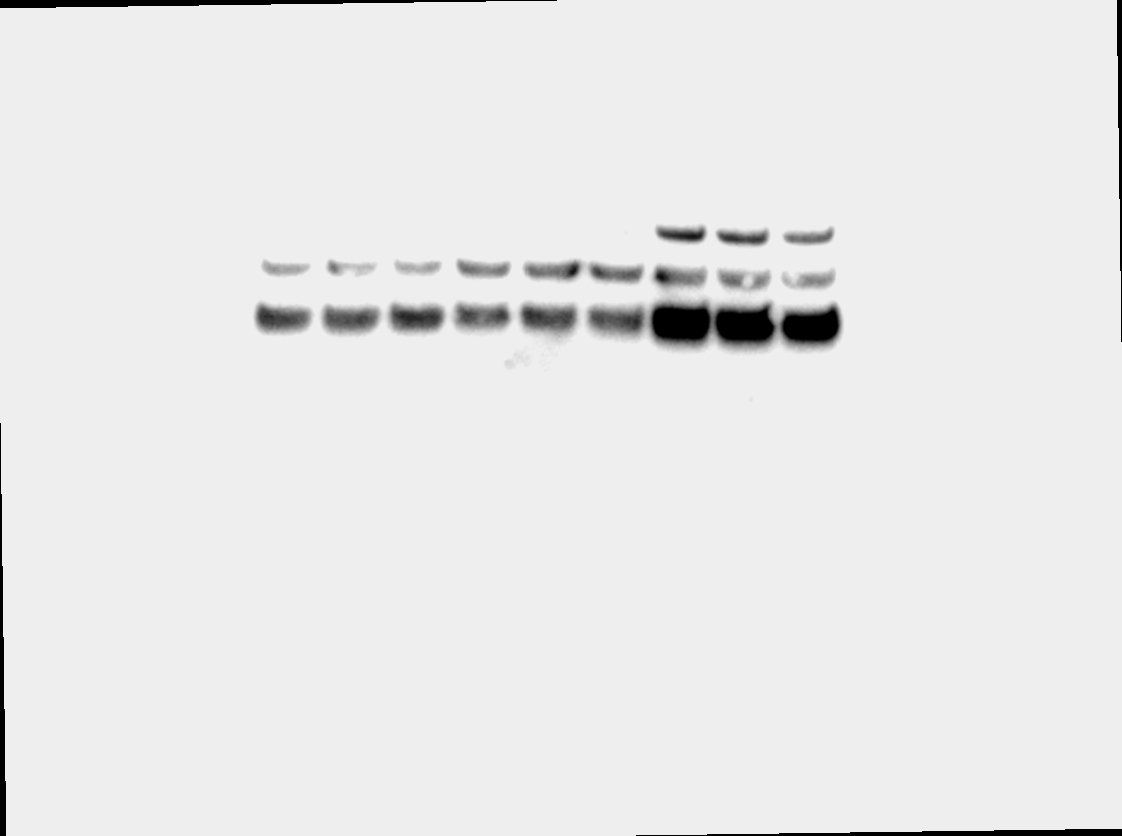

Supplement: Supplemental Information 6 [file peerj-11-16237-s006.zip › Raw data-WB Validation/Cal-27/Htra3-2.tif]

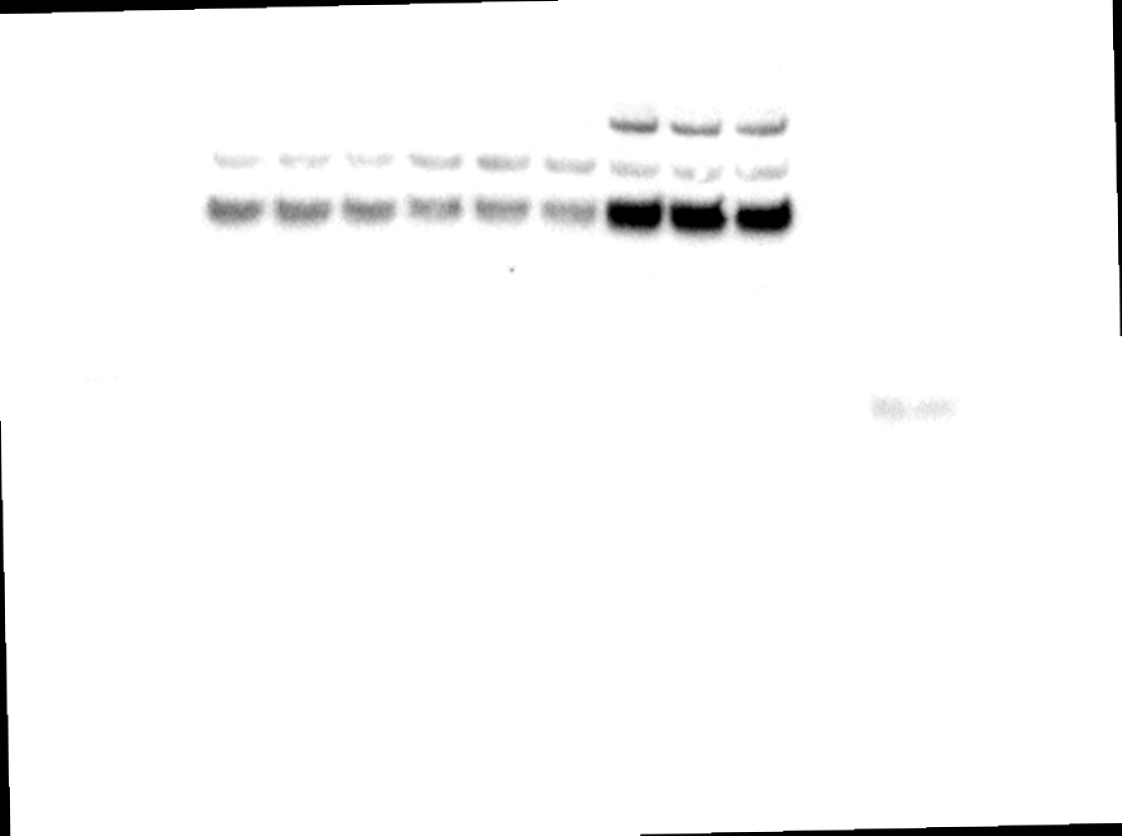

Supplement: Supplemental Information 6 [file peerj-11-16237-s006.zip › Raw data-WB Validation/Cal-27/Htra4-2.tif]

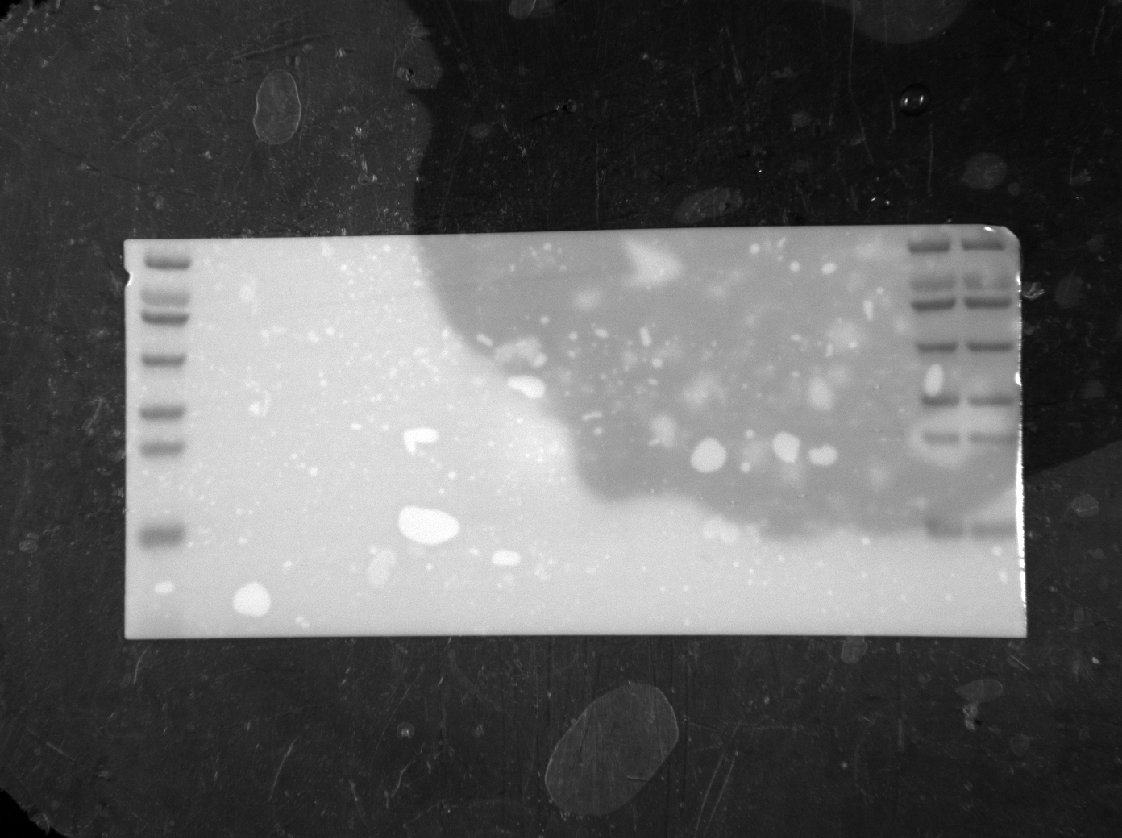

Supplement: Supplemental Information 6 [file peerj-11-16237-s006.zip › Raw data-WB Validation/Cal-27/htra2-marker.tif]

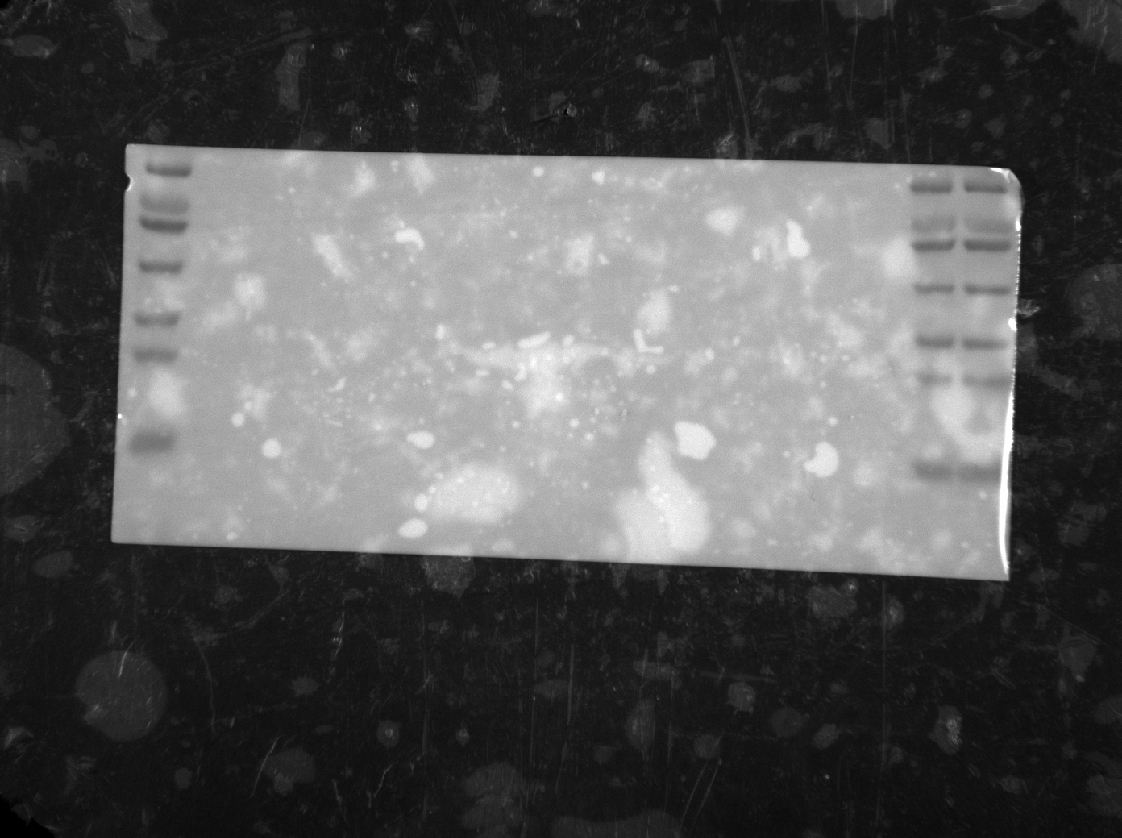

Supplement: Supplemental Information 6 [file peerj-11-16237-s006.zip › Raw data-WB Validation/Cal-27/htra3-marker.tif]

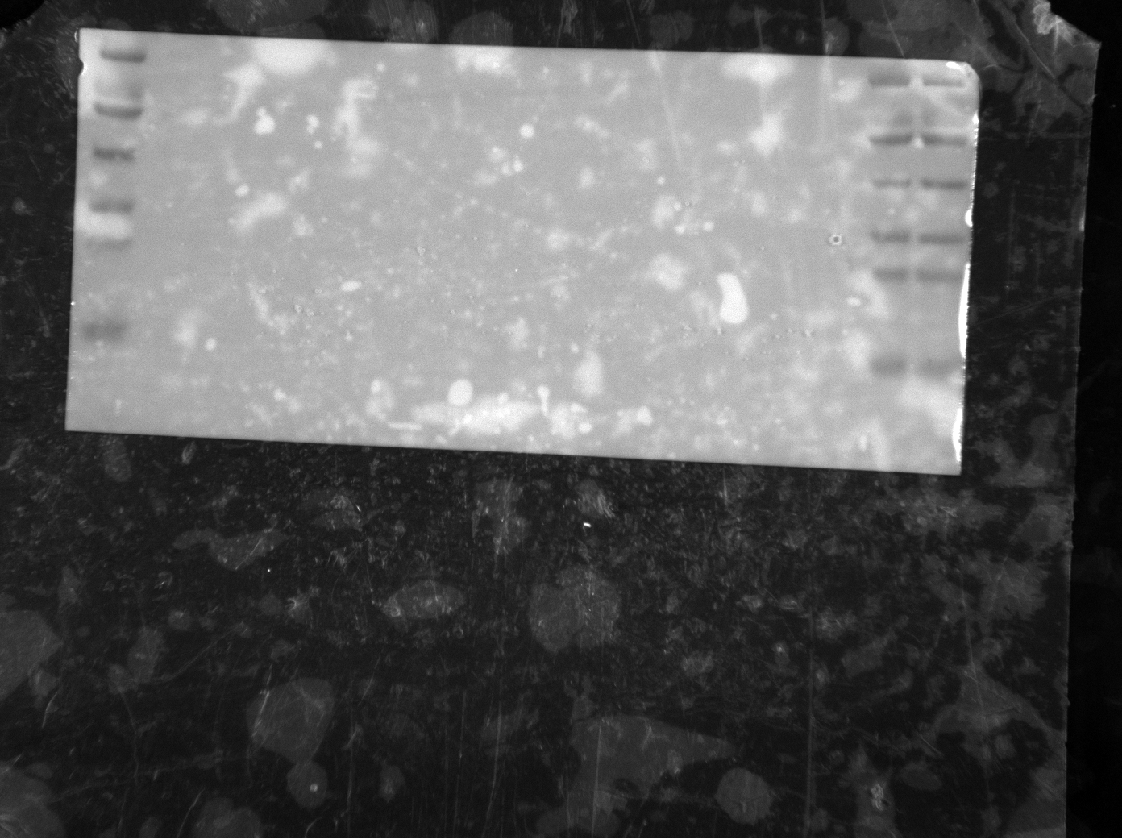

Supplement: Supplemental Information 6 [file peerj-11-16237-s006.zip › Raw data-WB Validation/Cal-27/htra4-marker.tif]

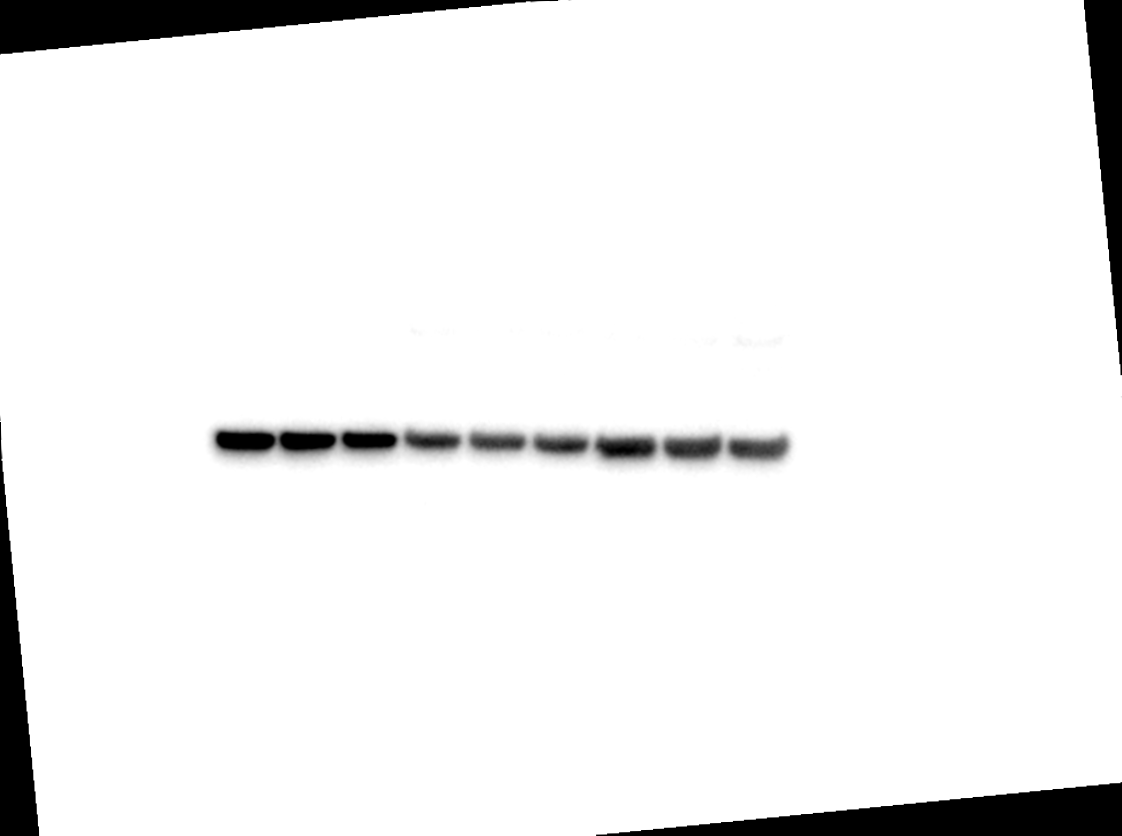

Supplement: Supplemental Information 6 [file peerj-11-16237-s006.zip › Raw data-WB Validation/FaDu/HTRA1-1.tif]

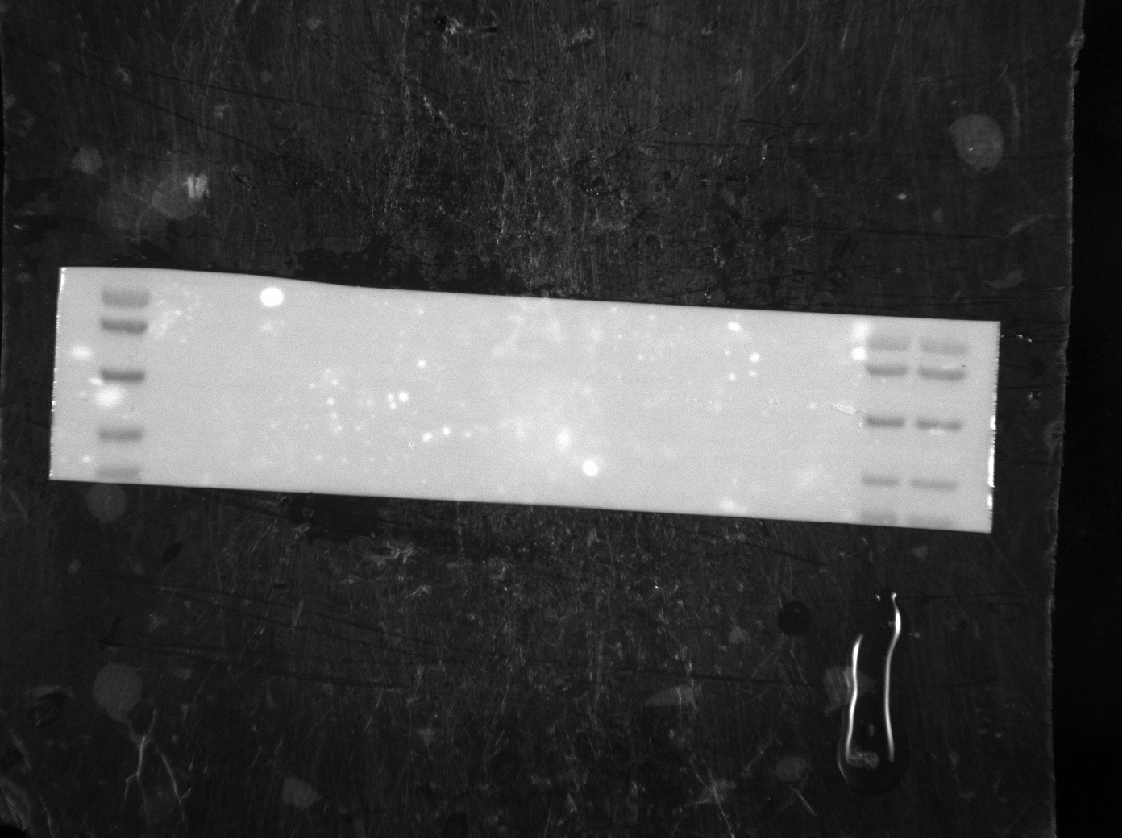

Supplement: Supplemental Information 6 [file peerj-11-16237-s006.zip › Raw data-WB Validation/FaDu/HTRA1-Marker.tif]

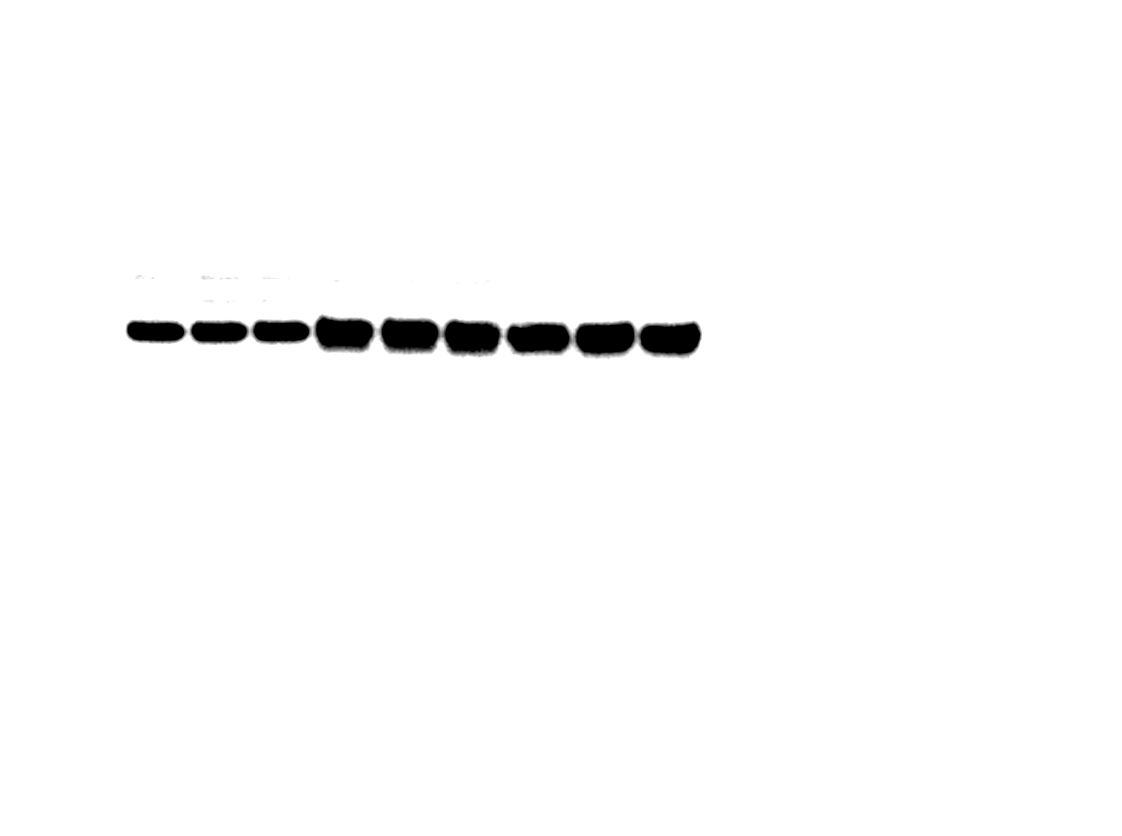

Supplement: Supplemental Information 6 [file peerj-11-16237-s006.zip › Raw data-WB Validation/FaDu/HTRA2-1.tif]

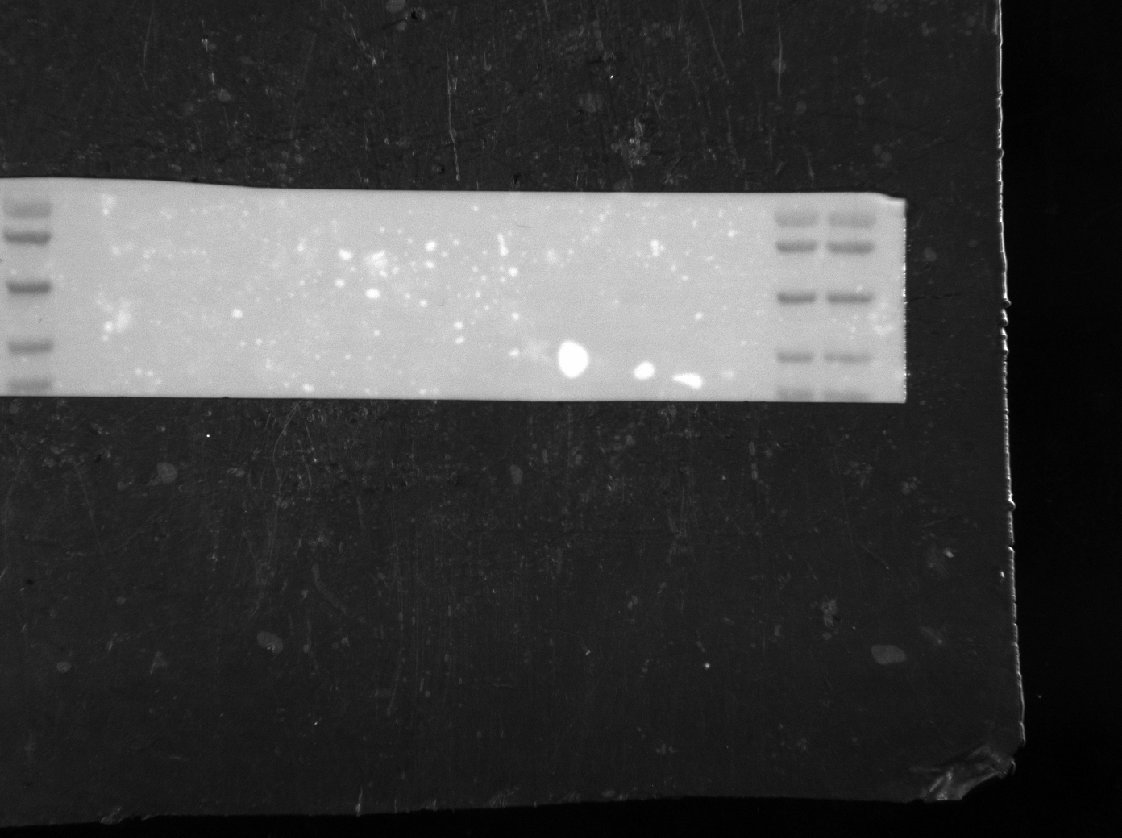

Supplement: Supplemental Information 6 [file peerj-11-16237-s006.zip › Raw data-WB Validation/FaDu/HTRA2-marker.tif]

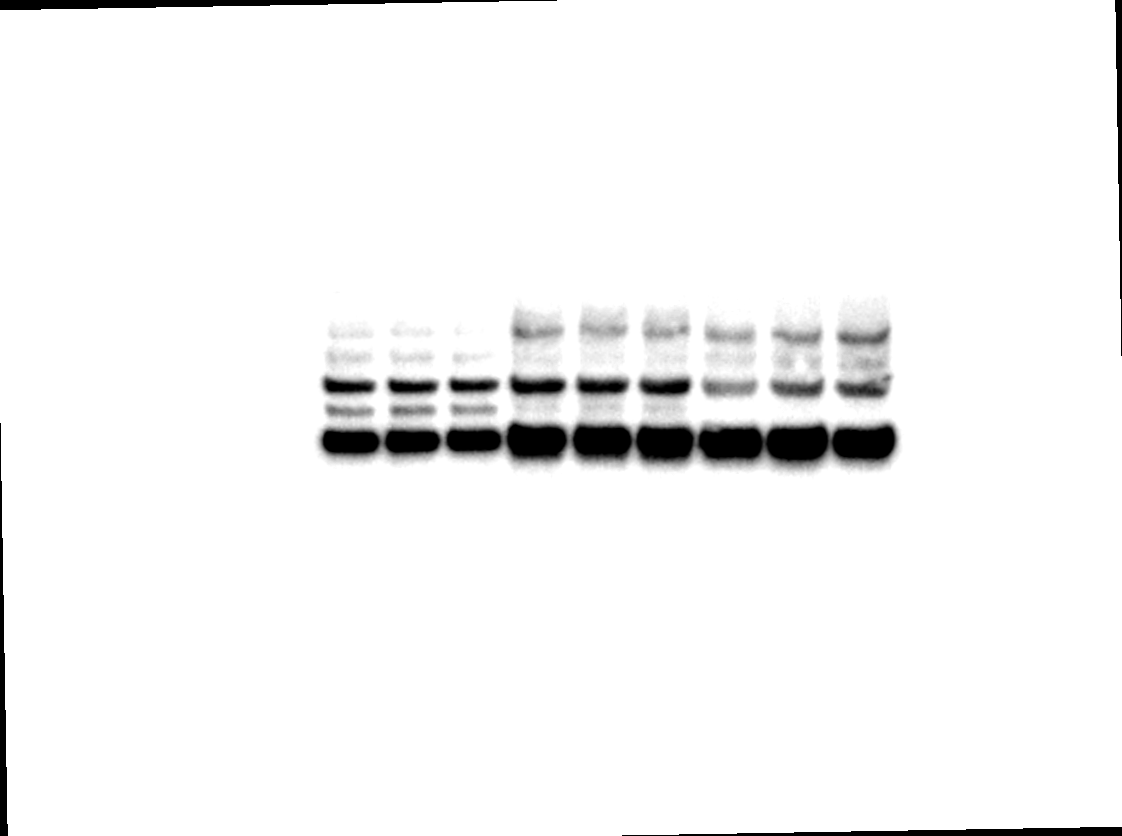

Supplement: Supplemental Information 6 [file peerj-11-16237-s006.zip › Raw data-WB Validation/FaDu/HTRA3-1.tif]

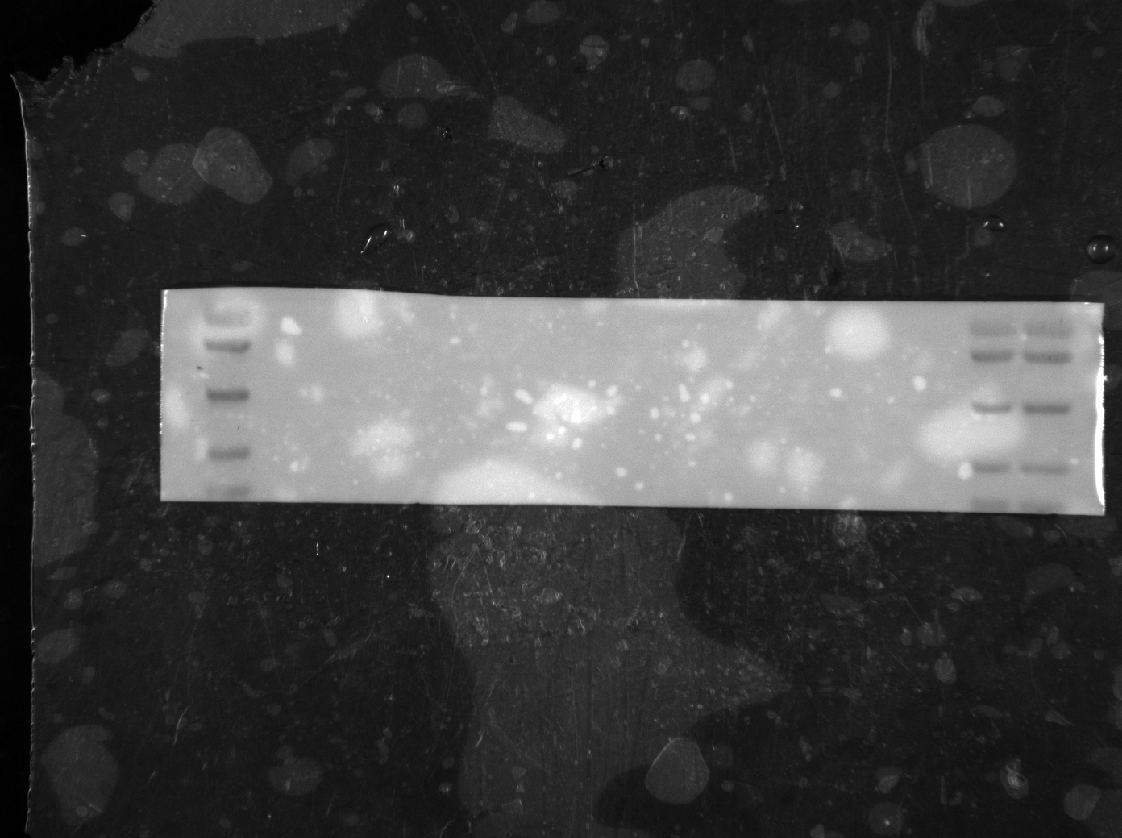

Supplement: Supplemental Information 6 [file peerj-11-16237-s006.zip › Raw data-WB Validation/FaDu/HTRA3-marker.tif]

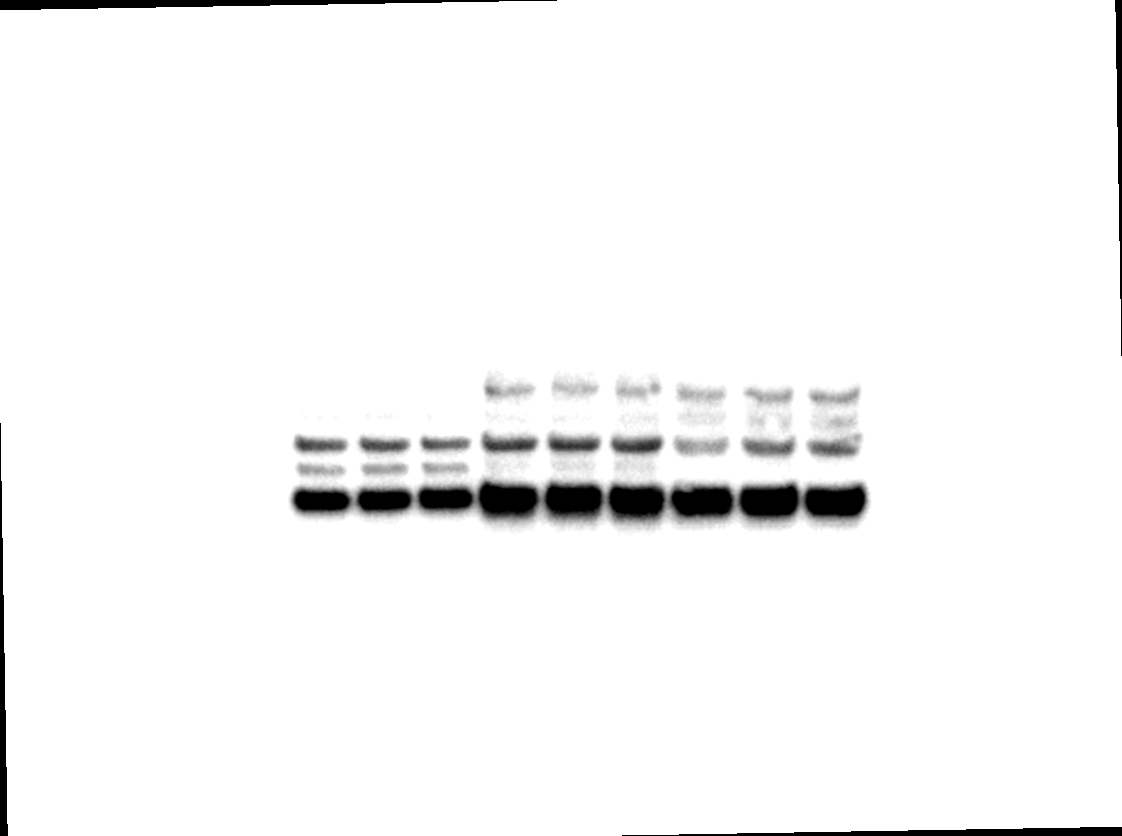

Supplement: Supplemental Information 6 [file peerj-11-16237-s006.zip › Raw data-WB Validation/FaDu/Htra4-2.tif]

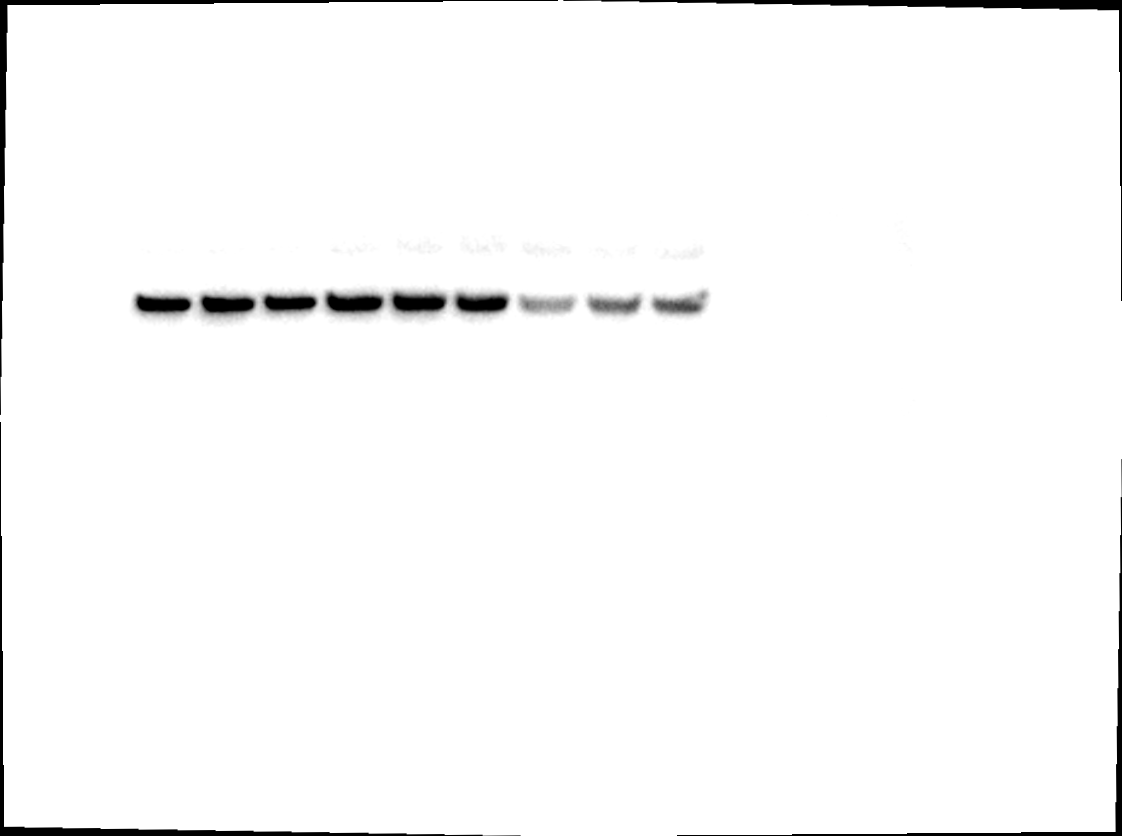

Supplement: Supplemental Information 6 [file peerj-11-16237-s006.zip › Raw data-WB Validation/FaDu/actin-6.tif]

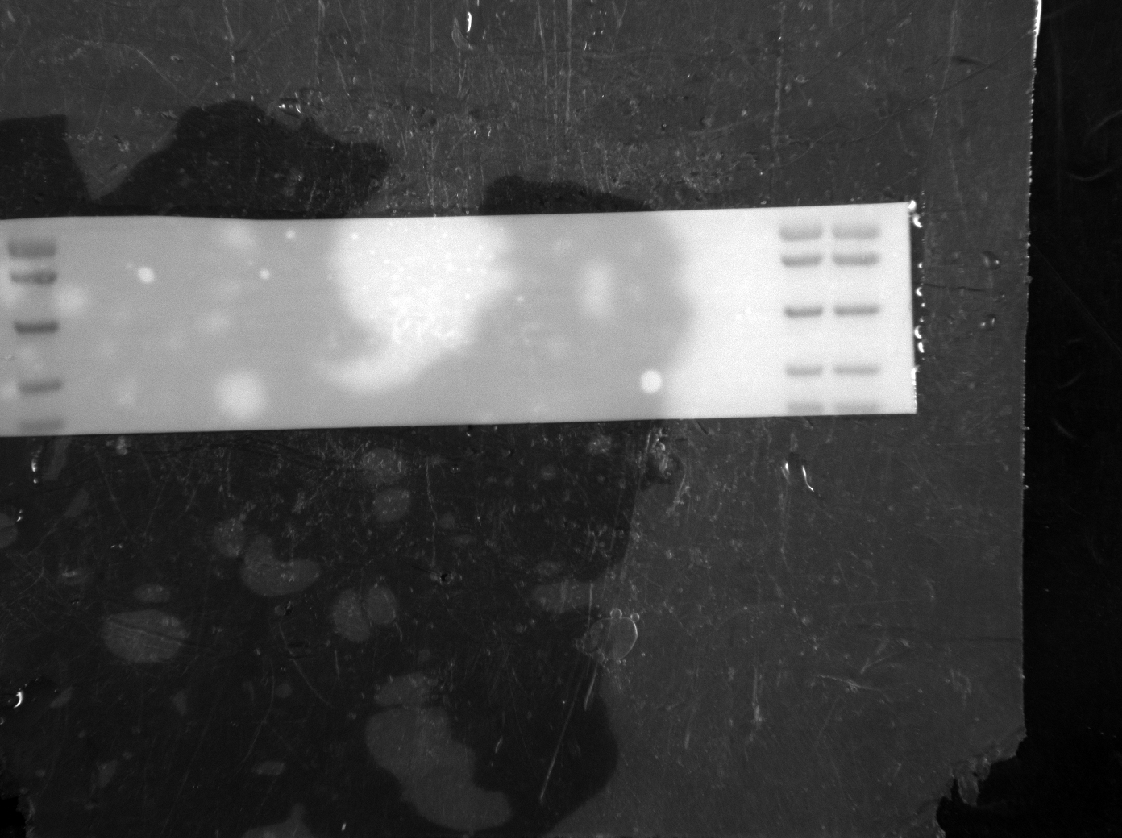

Supplement: Supplemental Information 6 [file peerj-11-16237-s006.zip › Raw data-WB Validation/FaDu/actin-Marker.tif]

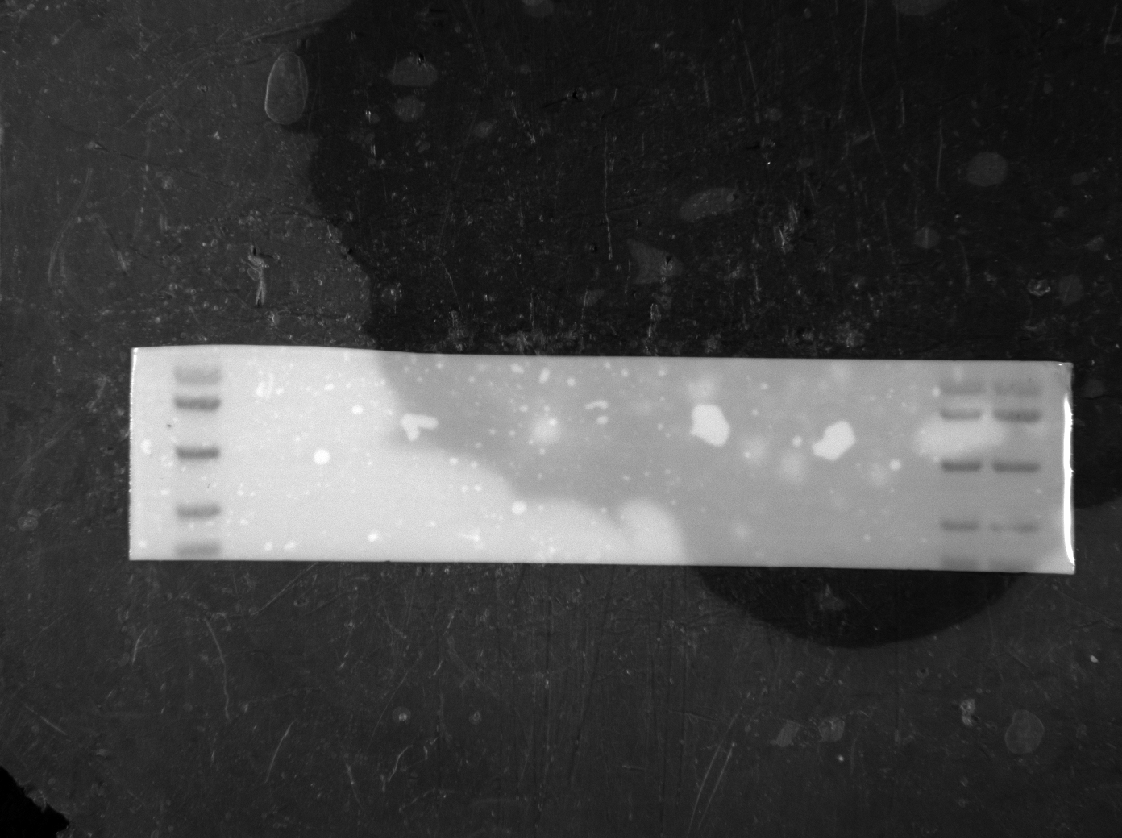

Supplement: Supplemental Information 6 [file peerj-11-16237-s006.zip › Raw data-WB Validation/FaDu/htra4-marker.tif]

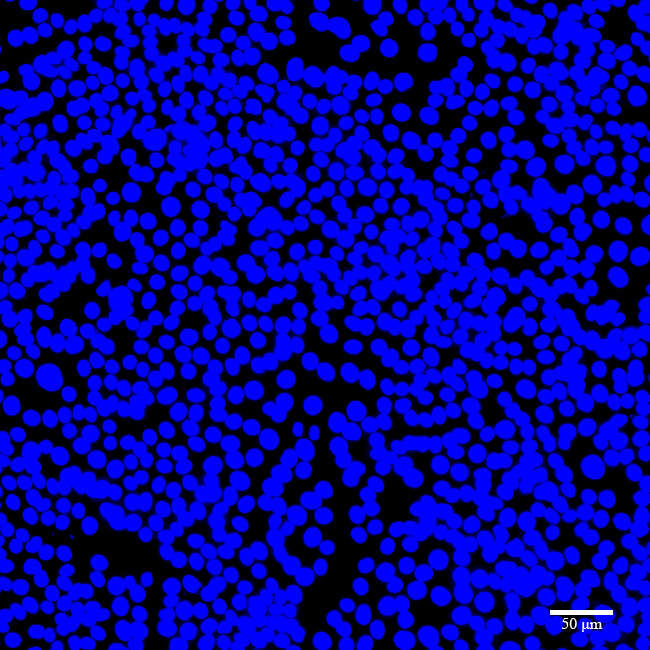

Supplement: Supplemental Information 9 [file peerj-11-16237-s009.zip › Raw data-Ki67-FAUD/fadu control/3blue.tif]

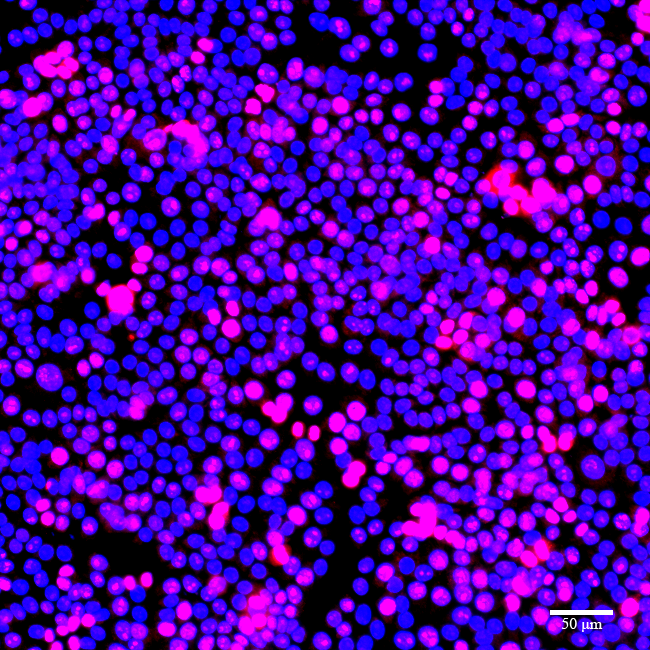

Supplement: Supplemental Information 9 [file peerj-11-16237-s009.zip › Raw data-Ki67-FAUD/fadu control/3merge.tif]

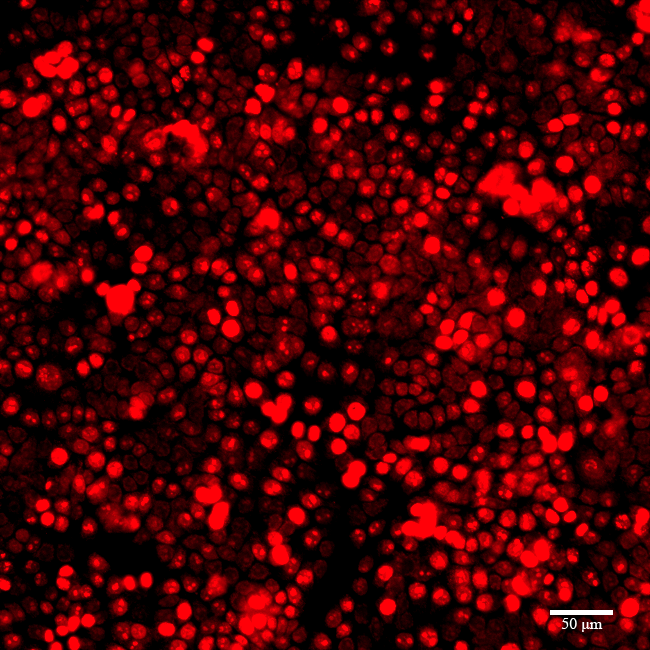

Supplement: Supplemental Information 9 [file peerj-11-16237-s009.zip › Raw data-Ki67-FAUD/fadu control/3red.tif]

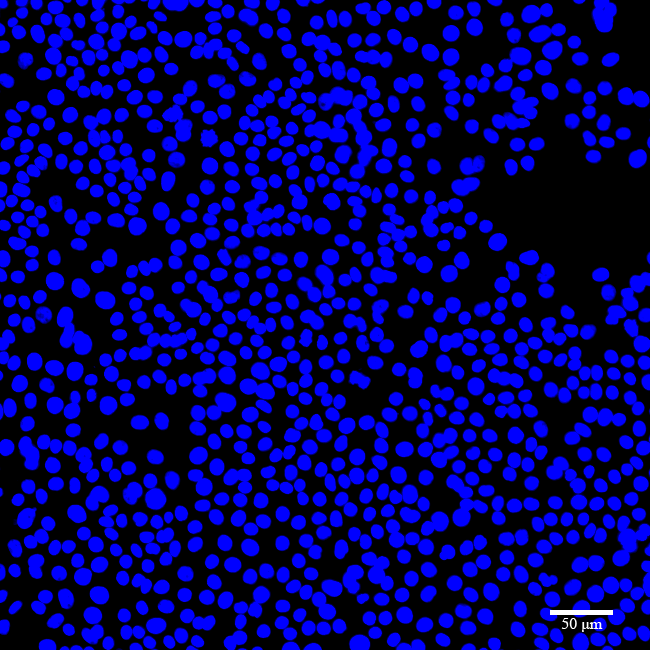

Supplement: Supplemental Information 9 [file peerj-11-16237-s009.zip › Raw data-Ki67-FAUD/fadu control/4blue.tif]

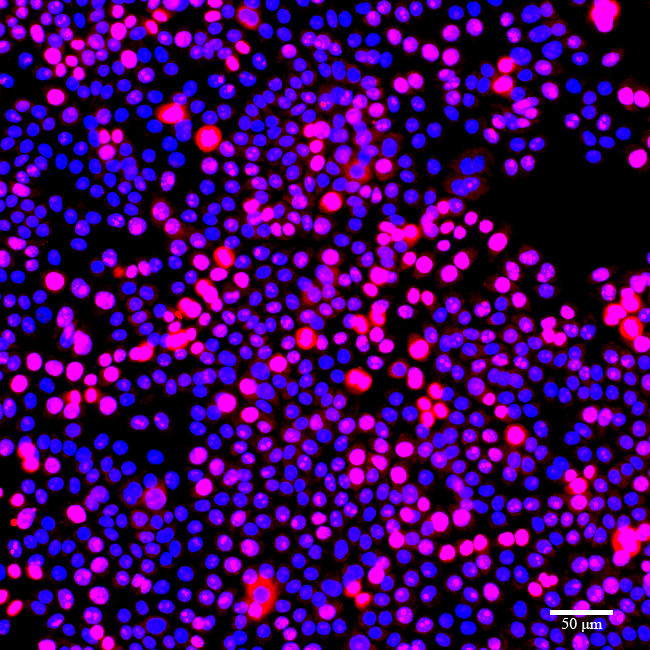

Supplement: Supplemental Information 9 [file peerj-11-16237-s009.zip › Raw data-Ki67-FAUD/fadu control/4merge.tif]

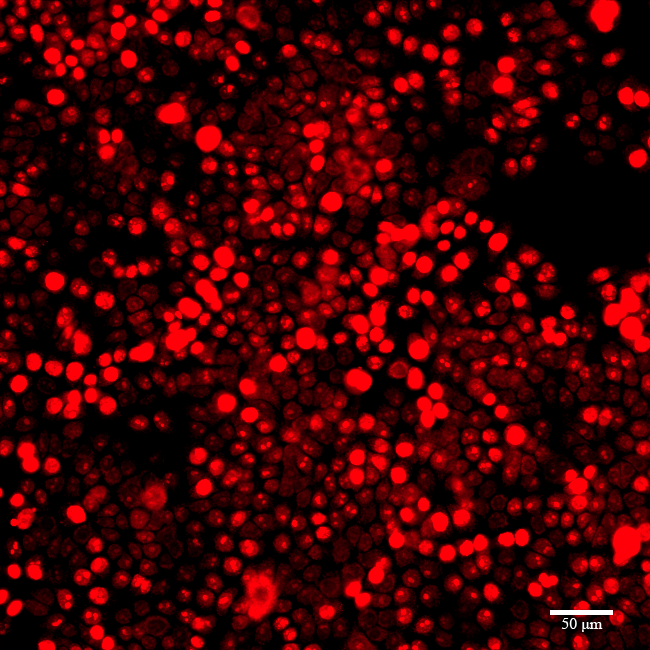

Supplement: Supplemental Information 9 [file peerj-11-16237-s009.zip › Raw data-Ki67-FAUD/fadu control/4red.tif]

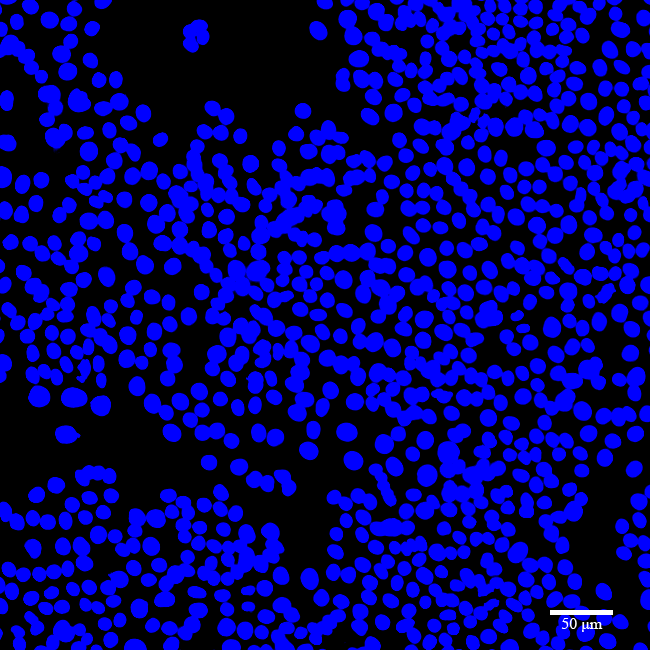

Supplement: Supplemental Information 9 [file peerj-11-16237-s009.zip › Raw data-Ki67-FAUD/fadu control/6blue.tif]

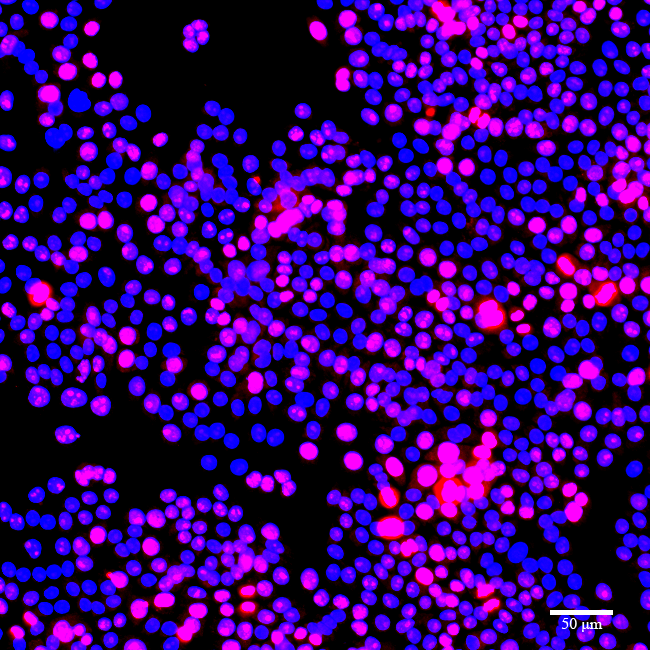

Supplement: Supplemental Information 9 [file peerj-11-16237-s009.zip › Raw data-Ki67-FAUD/fadu control/6merge.tif]

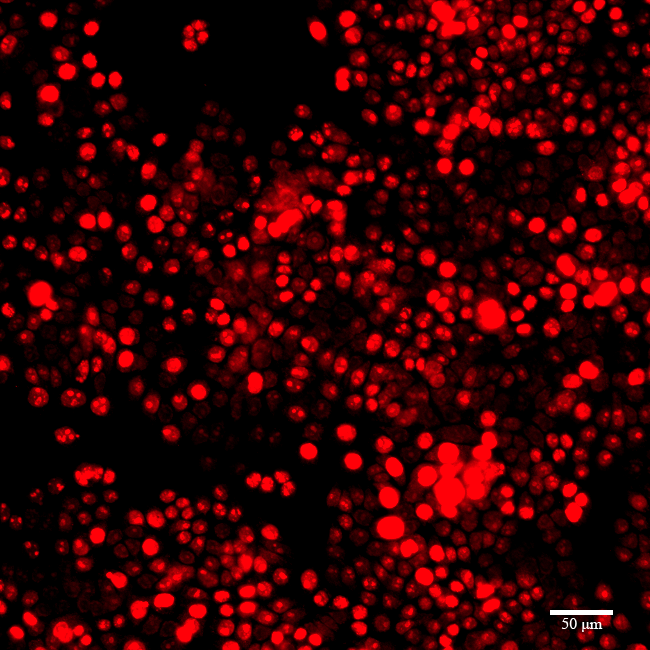

Supplement: Supplemental Information 9 [file peerj-11-16237-s009.zip › Raw data-Ki67-FAUD/fadu control/6red.tif]

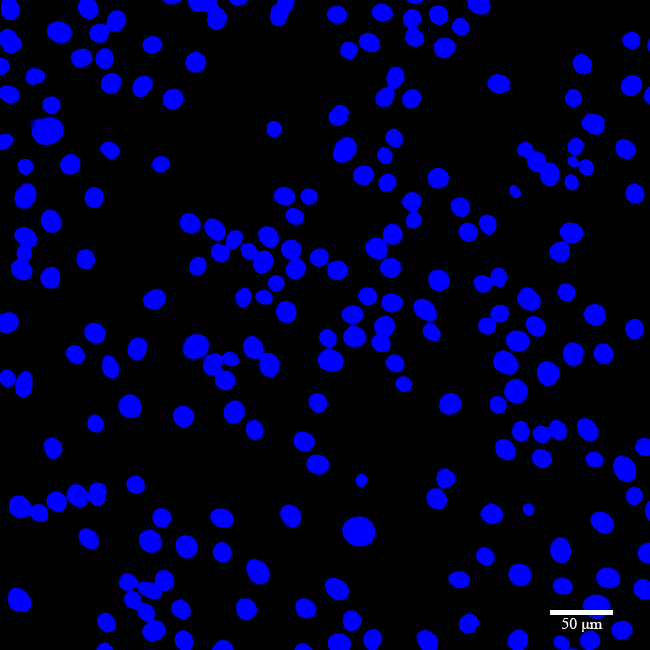

Supplement: Supplemental Information 9 [file peerj-11-16237-s009.zip › Raw data-Ki67-FAUD/fadu shHtrA3/2blue.tif]

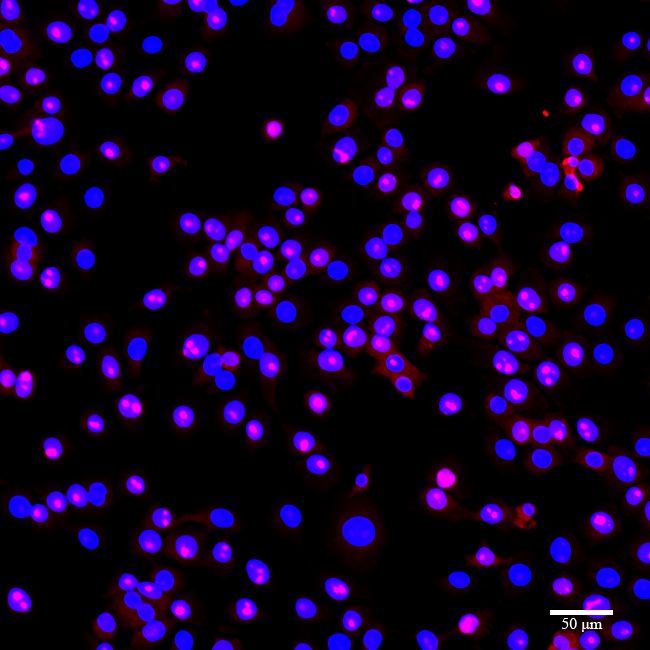

Supplement: Supplemental Information 9 [file peerj-11-16237-s009.zip › Raw data-Ki67-FAUD/fadu shHtrA3/2merge.tif]

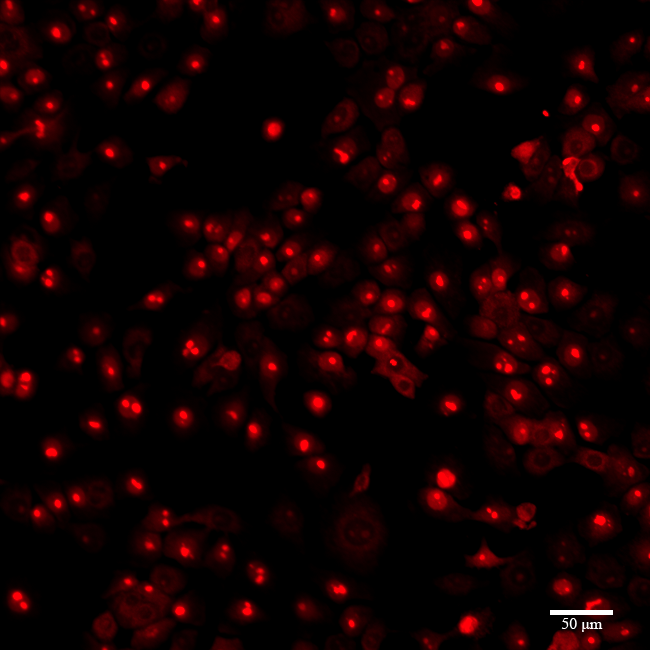

Supplement: Supplemental Information 9 [file peerj-11-16237-s009.zip › Raw data-Ki67-FAUD/fadu shHtrA3/2red.tif]

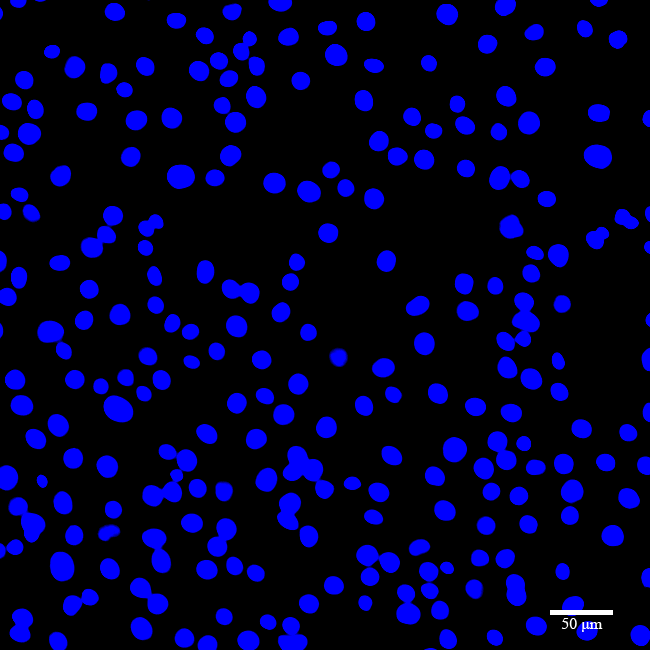

Supplement: Supplemental Information 9 [file peerj-11-16237-s009.zip › Raw data-Ki67-FAUD/fadu shHtrA3/5blue.tif]

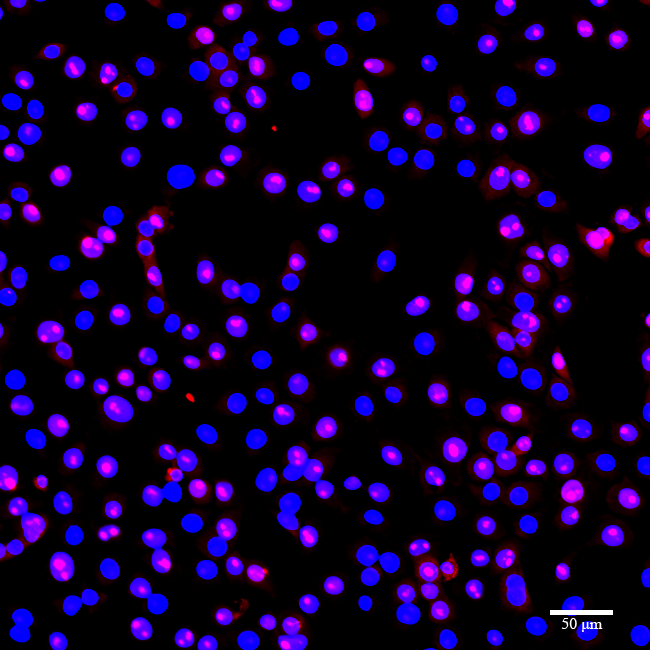

Supplement: Supplemental Information 9 [file peerj-11-16237-s009.zip › Raw data-Ki67-FAUD/fadu shHtrA3/5merge.tif]

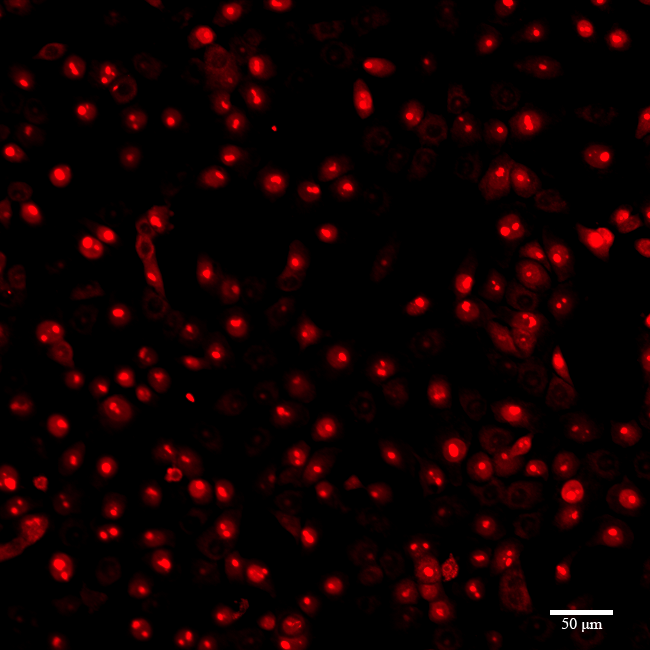

Supplement: Supplemental Information 9 [file peerj-11-16237-s009.zip › Raw data-Ki67-FAUD/fadu shHtrA3/5red.tif]

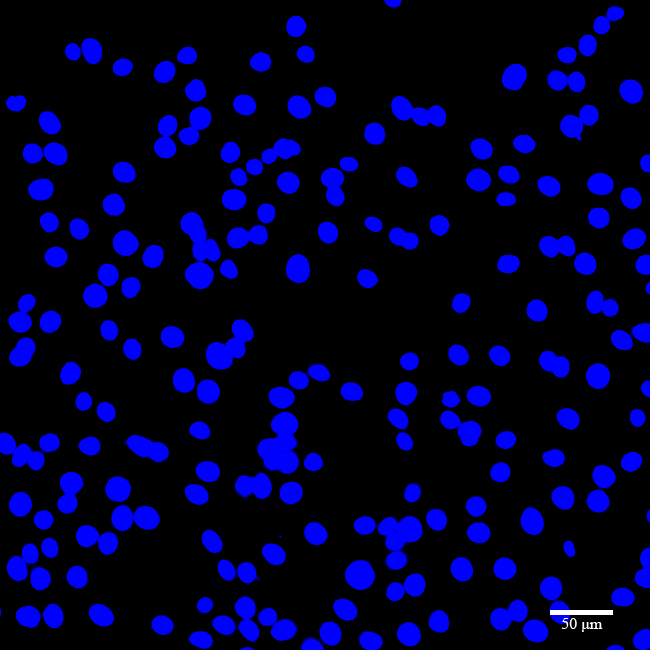

Supplement: Supplemental Information 9 [file peerj-11-16237-s009.zip › Raw data-Ki67-FAUD/fadu shHtrA3/6blue.tif]

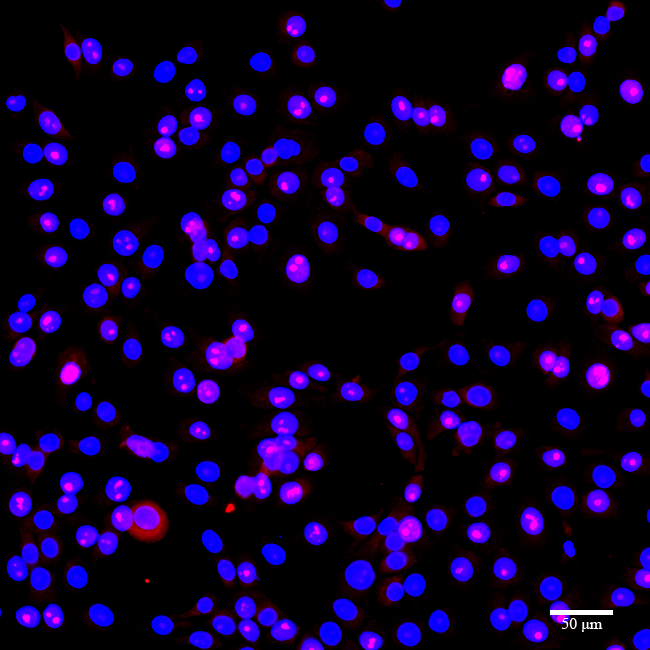

Supplement: Supplemental Information 9 [file peerj-11-16237-s009.zip › Raw data-Ki67-FAUD/fadu shHtrA3/6merge.tif]

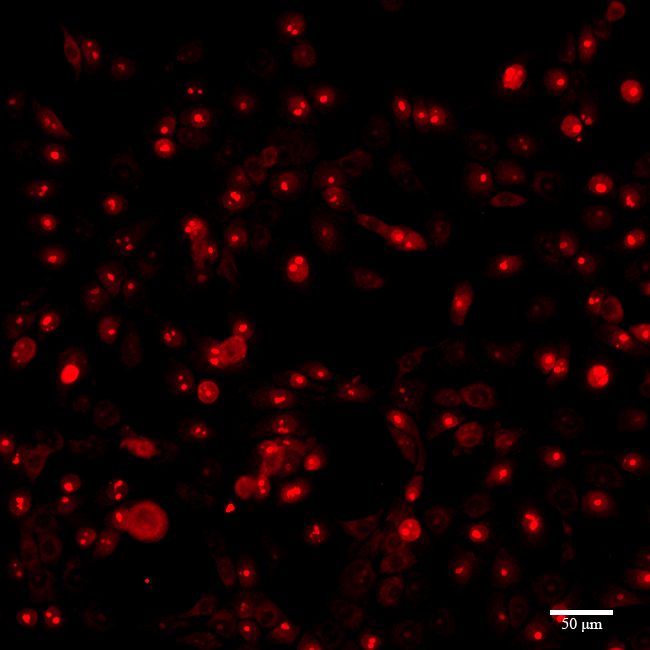

Supplement: Supplemental Information 9 [file peerj-11-16237-s009.zip › Raw data-Ki67-FAUD/fadu shHtrA3/6red.tif]

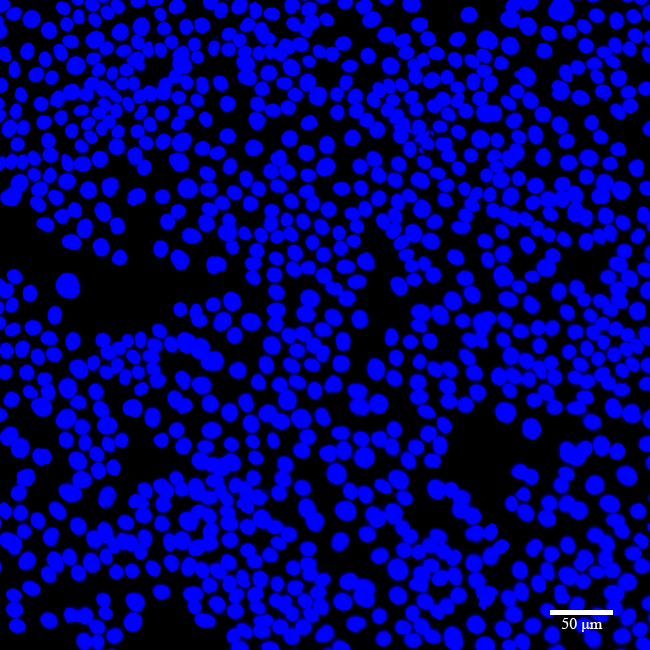

Supplement: Supplemental Information 9 [file peerj-11-16237-s009.zip › Raw data-Ki67-FAUD/fadu shNC/1blue.tif]

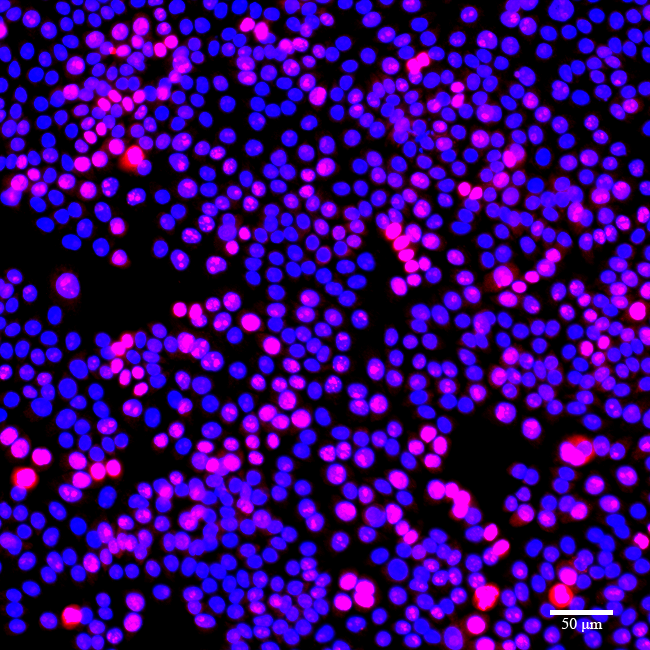

Supplement: Supplemental Information 9 [file peerj-11-16237-s009.zip › Raw data-Ki67-FAUD/fadu shNC/1merge.tif]

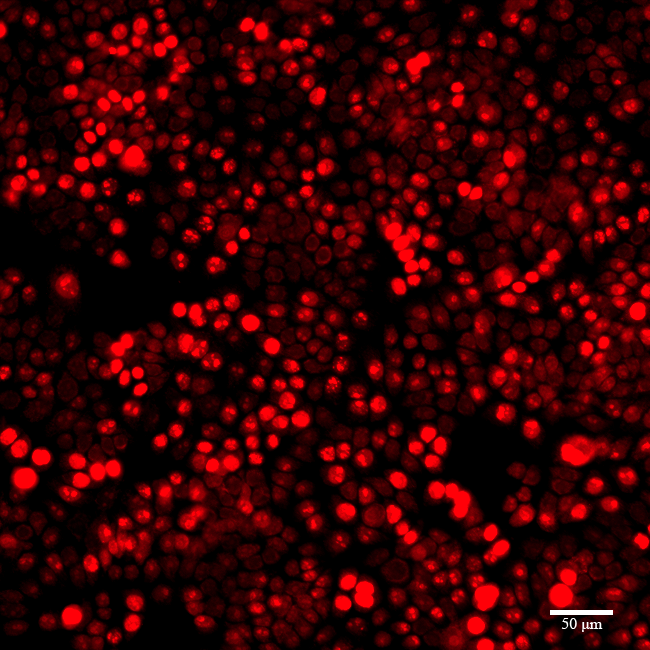

Supplement: Supplemental Information 9 [file peerj-11-16237-s009.zip › Raw data-Ki67-FAUD/fadu shNC/1red.tif]

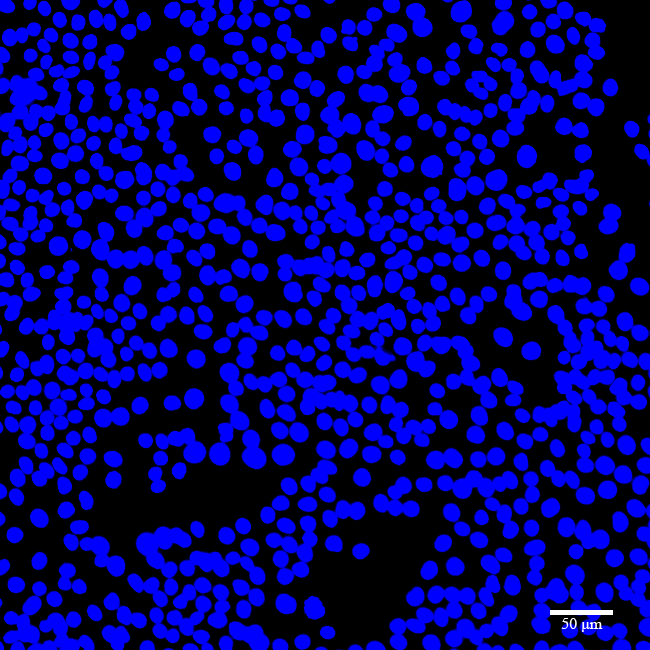

Supplement: Supplemental Information 9 [file peerj-11-16237-s009.zip › Raw data-Ki67-FAUD/fadu shNC/2blue.tif]

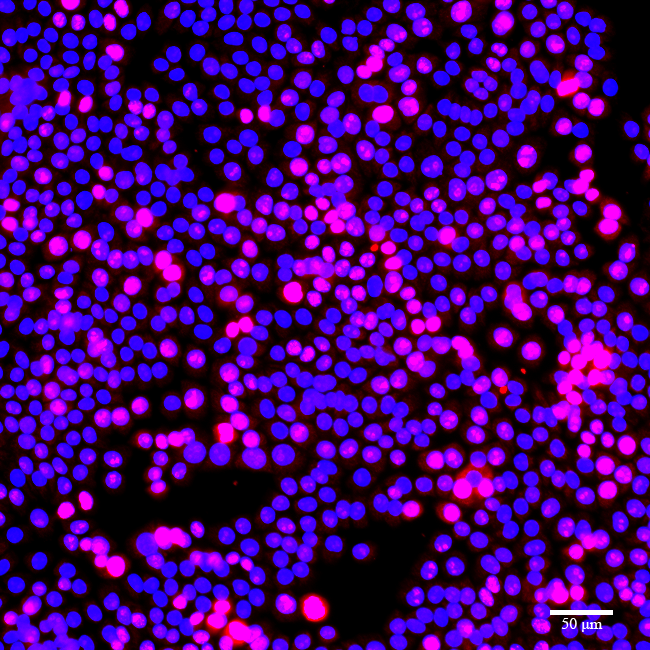

Supplement: Supplemental Information 9 [file peerj-11-16237-s009.zip › Raw data-Ki67-FAUD/fadu shNC/2merge.tif]

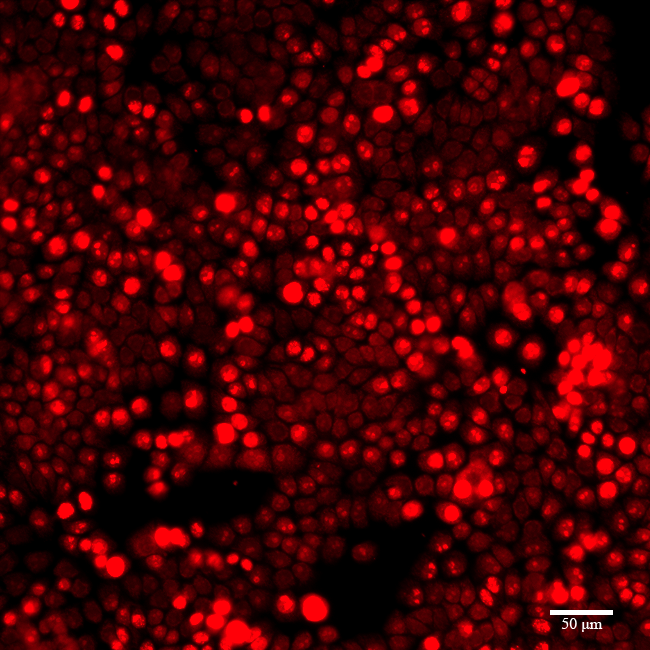

Supplement: Supplemental Information 9 [file peerj-11-16237-s009.zip › Raw data-Ki67-FAUD/fadu shNC/2red.tif]

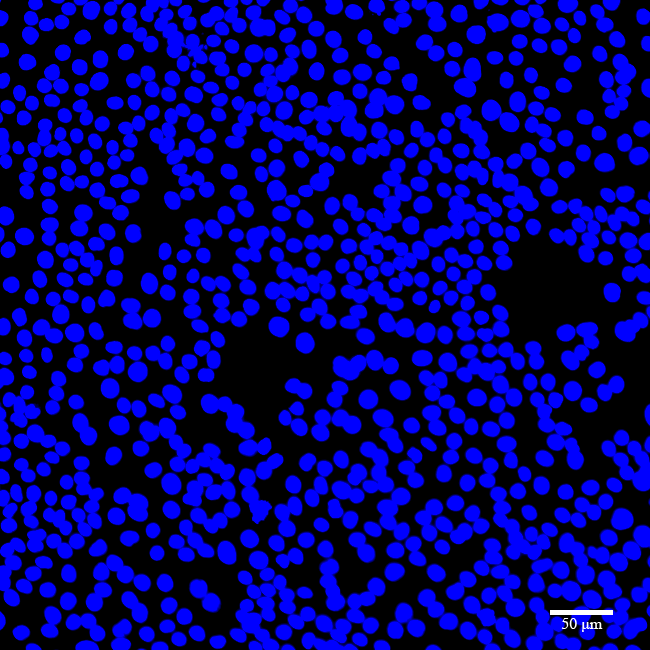

Supplement: Supplemental Information 9 [file peerj-11-16237-s009.zip › Raw data-Ki67-FAUD/fadu shNC/3blue.tif]

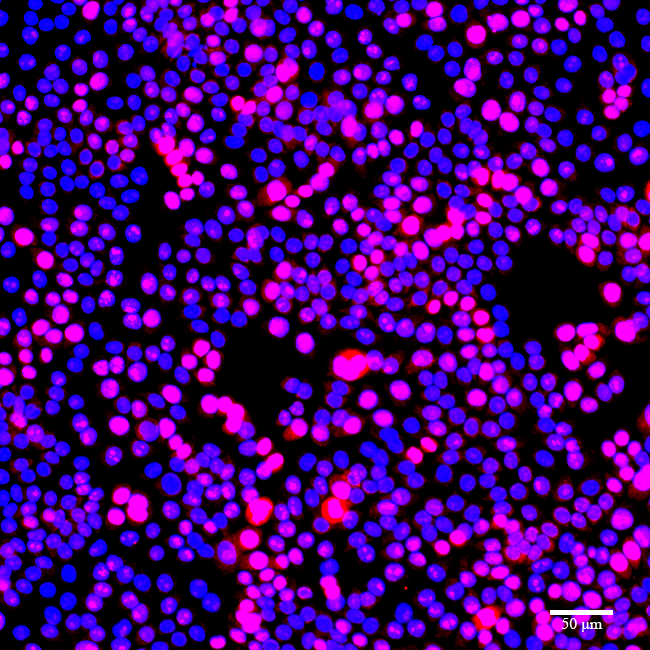

Supplement: Supplemental Information 9 [file peerj-11-16237-s009.zip › Raw data-Ki67-FAUD/fadu shNC/3merge.tif]

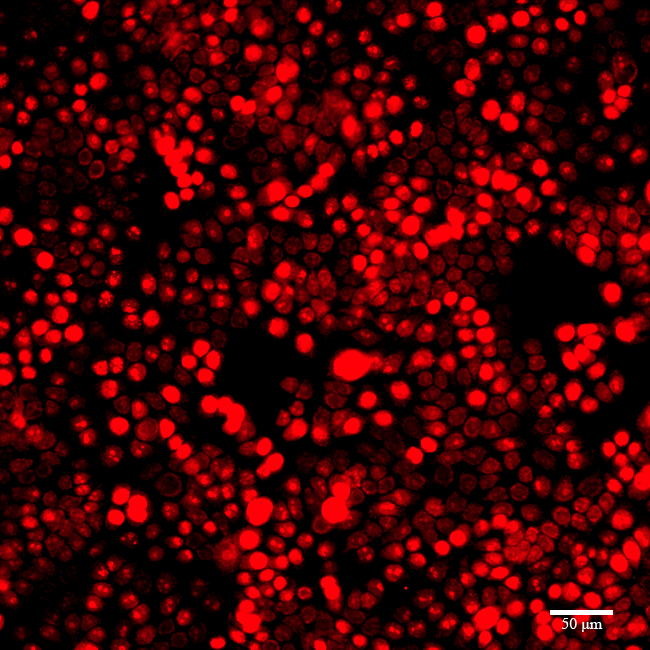

Supplement: Supplemental Information 9 [file peerj-11-16237-s009.zip › Raw data-Ki67-FAUD/fadu shNC/3red.tif]

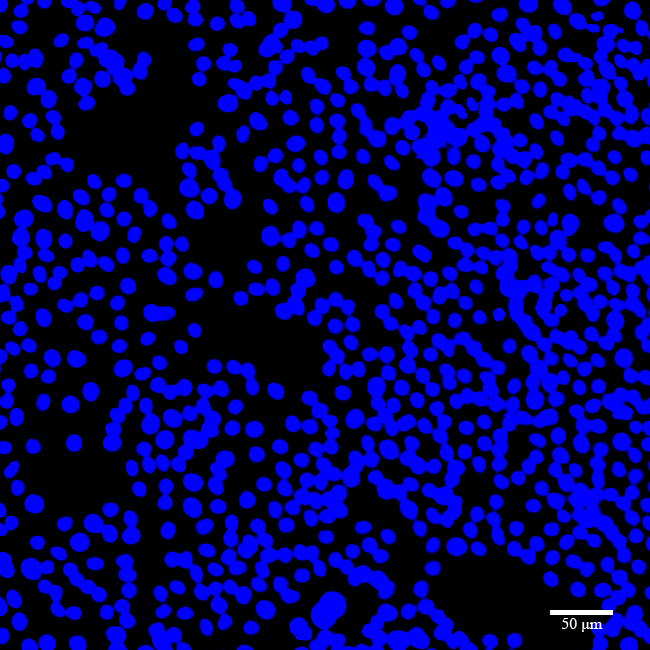

Supplement: Supplemental Information 10 [file peerj-11-16237-s010.zip › Raw data-Ki67-CAL27/cal27 control/3blue.tif]

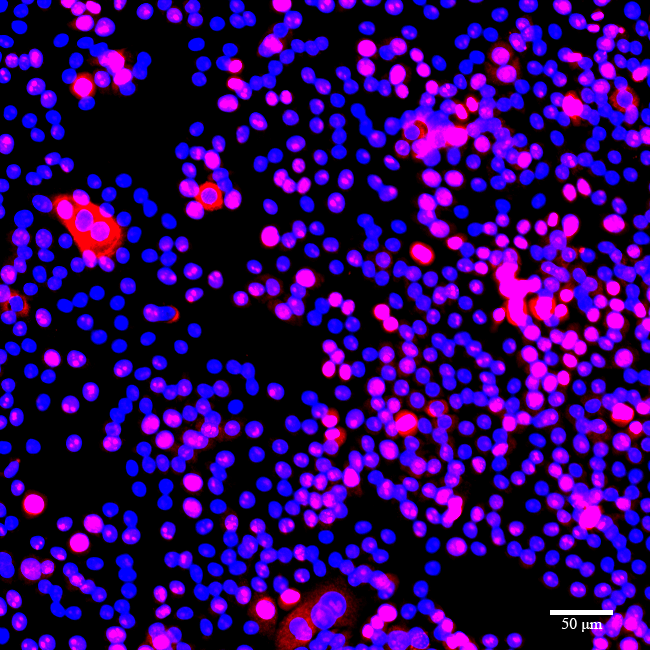

Supplement: Supplemental Information 10 [file peerj-11-16237-s010.zip › Raw data-Ki67-CAL27/cal27 control/3merge.tif]

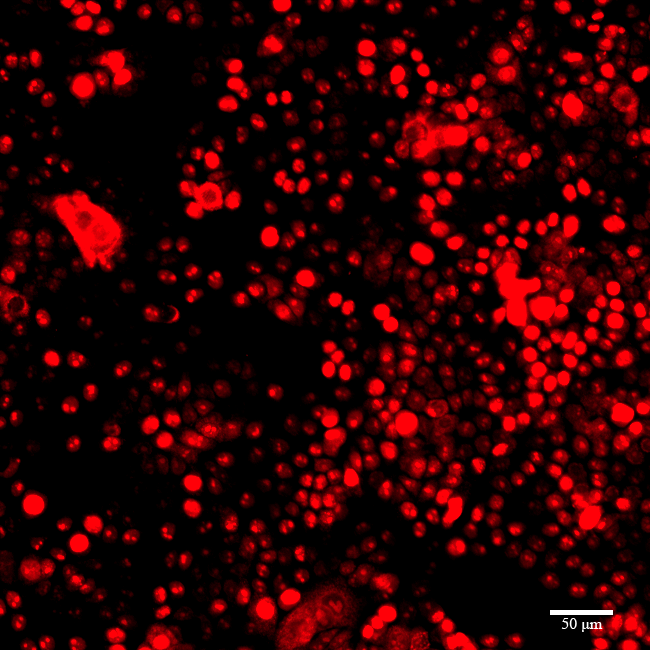

Supplement: Supplemental Information 10 [file peerj-11-16237-s010.zip › Raw data-Ki67-CAL27/cal27 control/3red.tif]

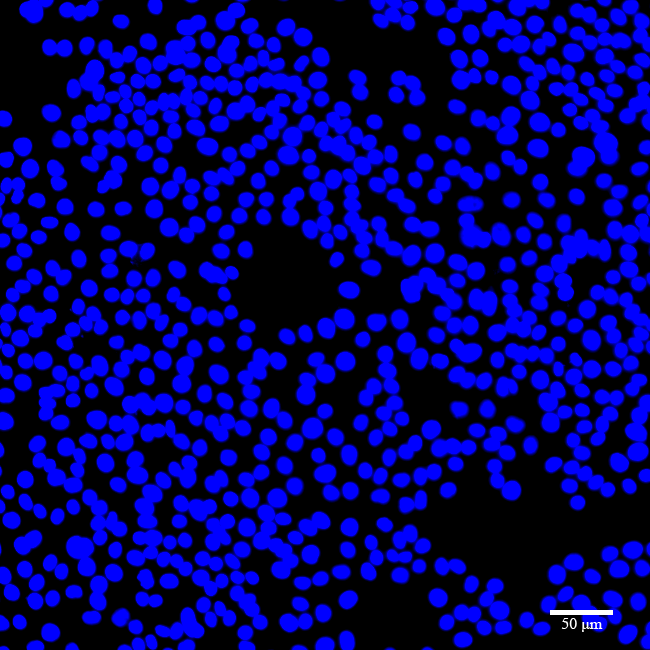

Supplement: Supplemental Information 10 [file peerj-11-16237-s010.zip › Raw data-Ki67-CAL27/cal27 control/4blue.tif]

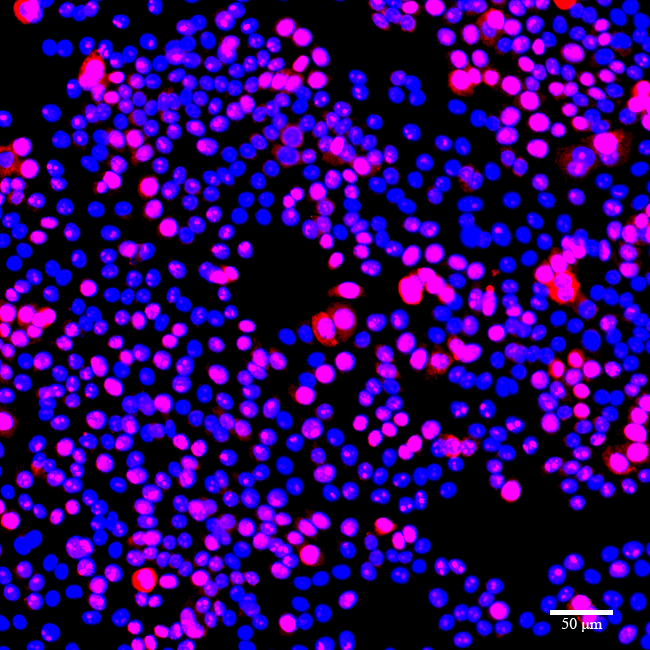

Supplement: Supplemental Information 10 [file peerj-11-16237-s010.zip › Raw data-Ki67-CAL27/cal27 control/4merge.tif]

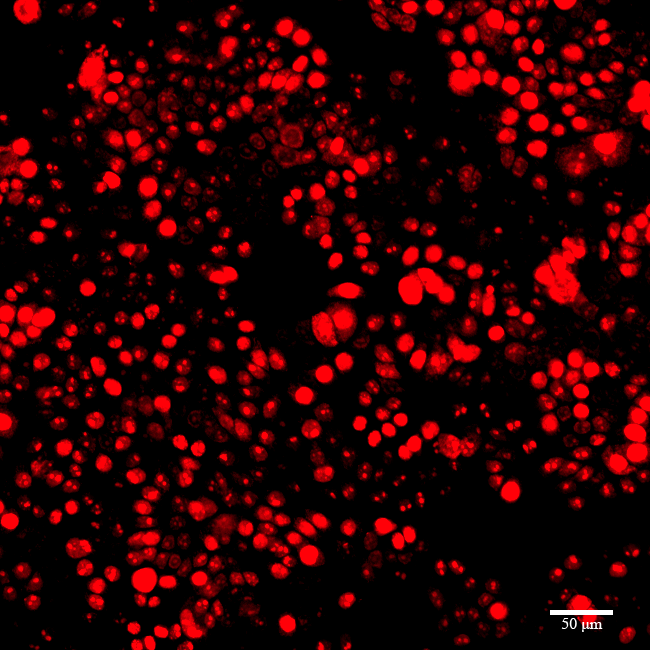

Supplement: Supplemental Information 10 [file peerj-11-16237-s010.zip › Raw data-Ki67-CAL27/cal27 control/4red.tif]

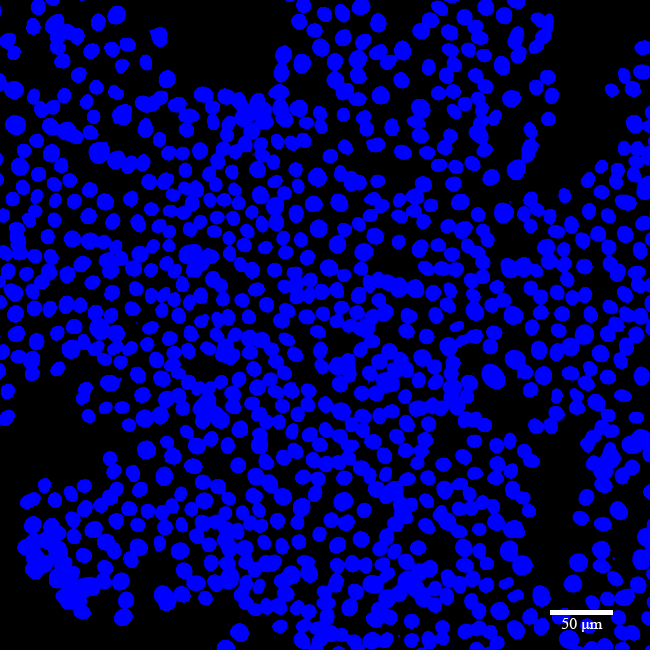

Supplement: Supplemental Information 10 [file peerj-11-16237-s010.zip › Raw data-Ki67-CAL27/cal27 control/6blue.tif]

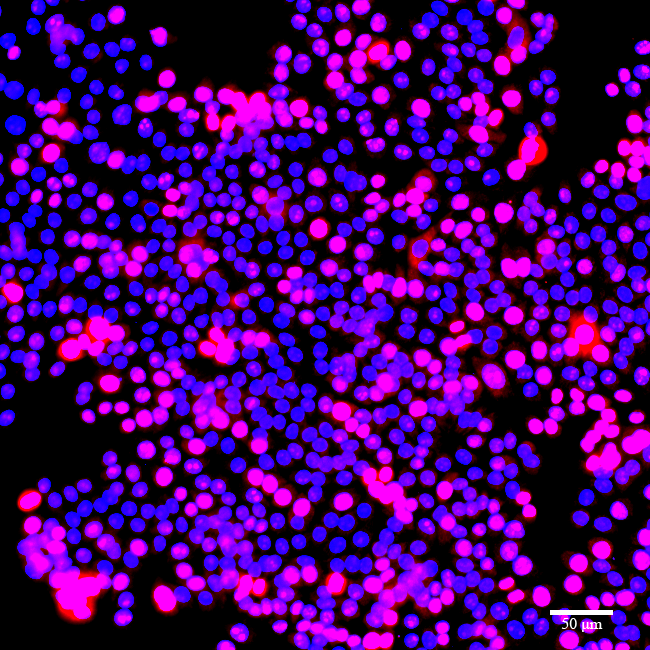

Supplement: Supplemental Information 10 [file peerj-11-16237-s010.zip › Raw data-Ki67-CAL27/cal27 control/6merge.tif]

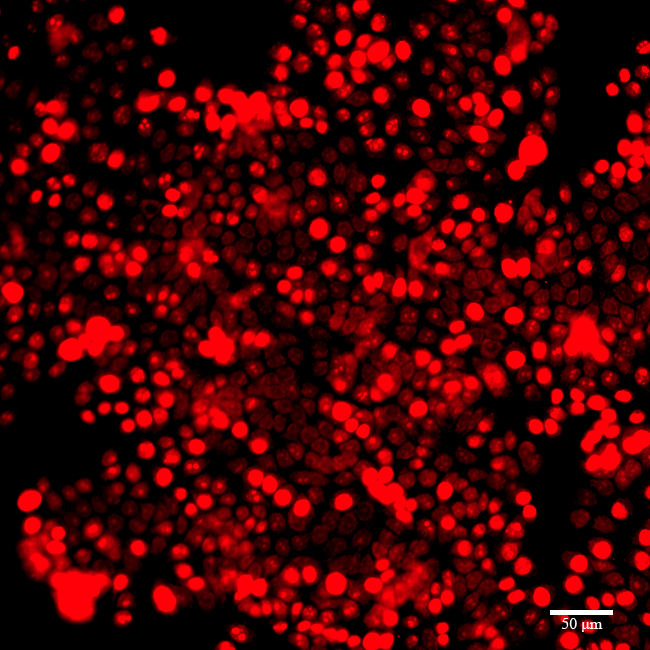

Supplement: Supplemental Information 10 [file peerj-11-16237-s010.zip › Raw data-Ki67-CAL27/cal27 control/6red.tif]

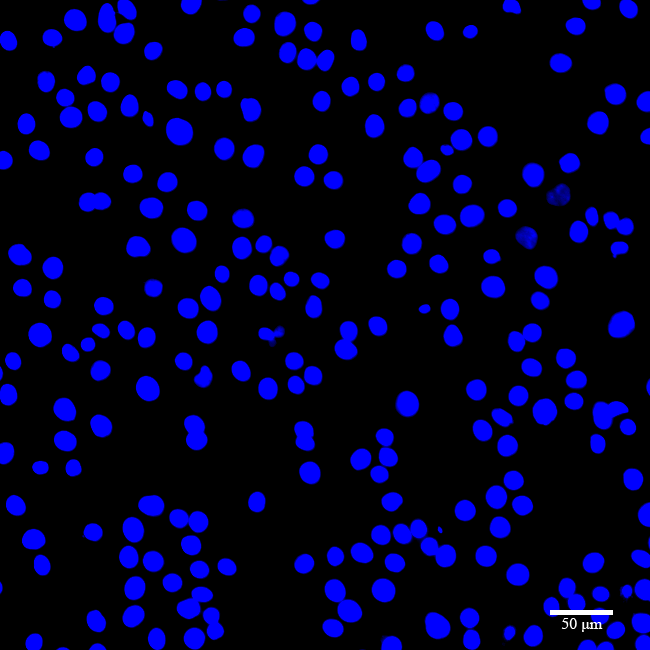

Supplement: Supplemental Information 10 [file peerj-11-16237-s010.zip › Raw data-Ki67-CAL27/cal27 shHtrA3/2blue.tif]

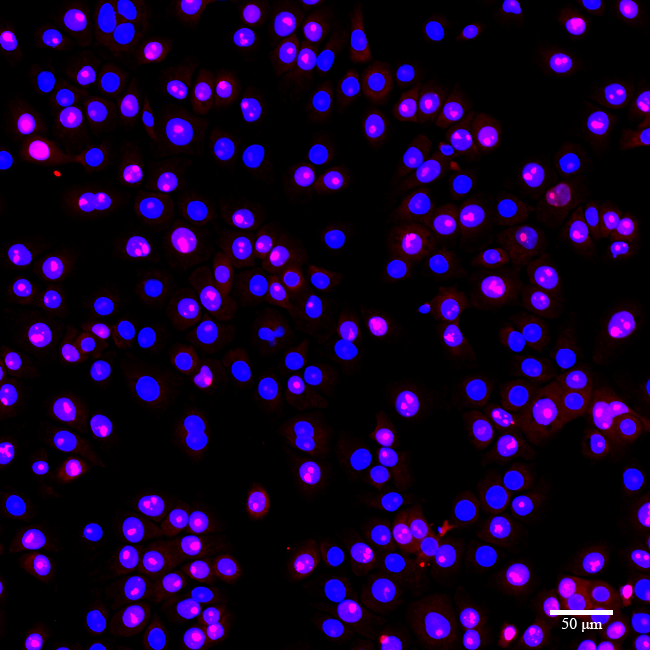

Supplement: Supplemental Information 10 [file peerj-11-16237-s010.zip › Raw data-Ki67-CAL27/cal27 shHtrA3/2merge.tif]

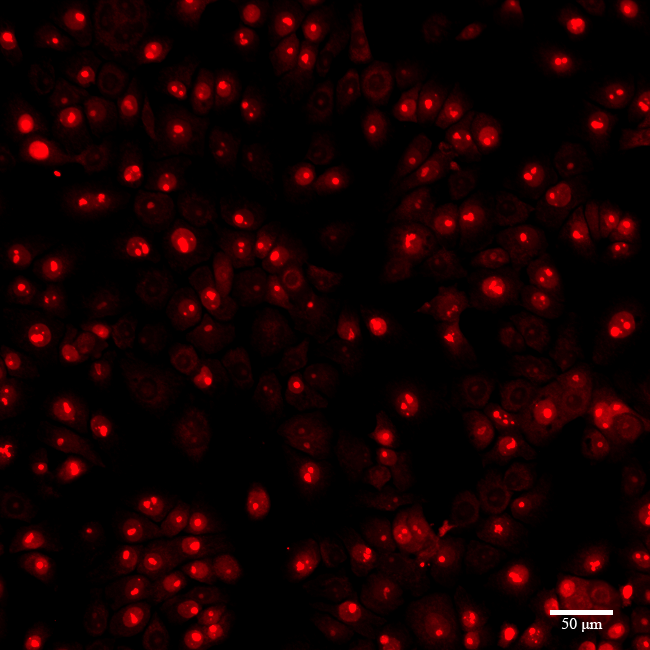

Supplement: Supplemental Information 10 [file peerj-11-16237-s010.zip › Raw data-Ki67-CAL27/cal27 shHtrA3/2red.tif]

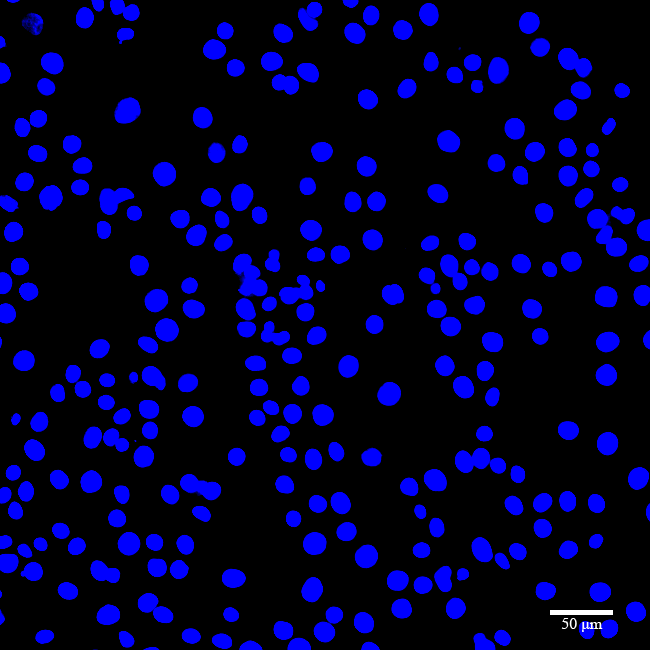

Supplement: Supplemental Information 10 [file peerj-11-16237-s010.zip › Raw data-Ki67-CAL27/cal27 shHtrA3/3blue.tif]

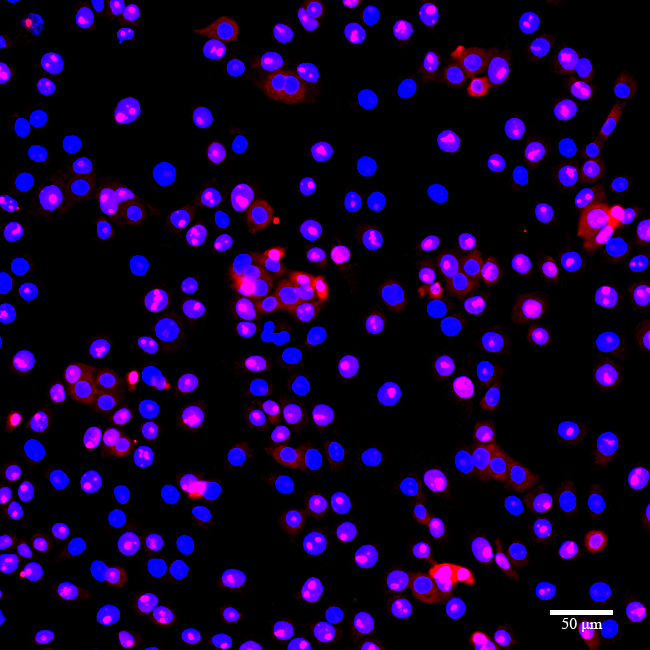

Supplement: Supplemental Information 10 [file peerj-11-16237-s010.zip › Raw data-Ki67-CAL27/cal27 shHtrA3/3merge.tif]

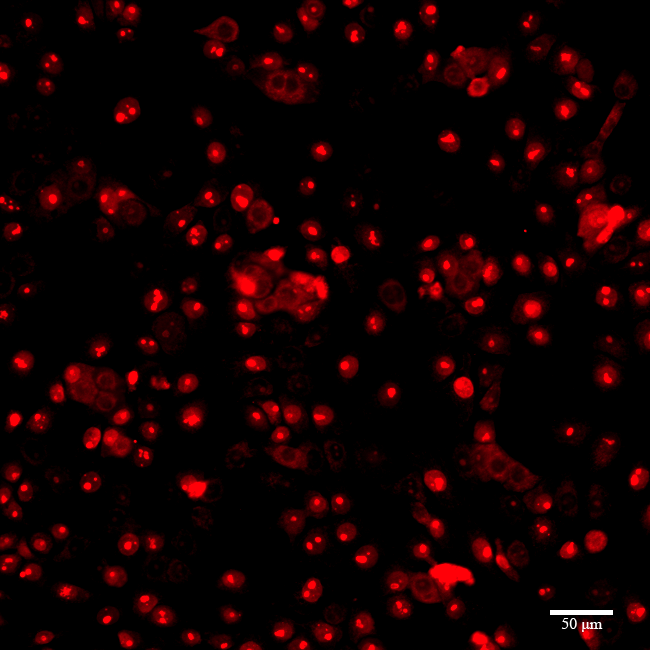

Supplement: Supplemental Information 10 [file peerj-11-16237-s010.zip › Raw data-Ki67-CAL27/cal27 shHtrA3/3red.tif]

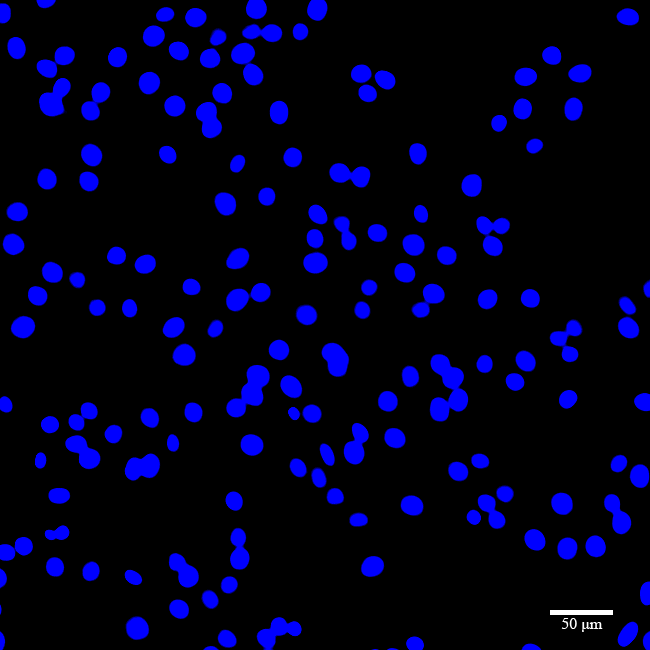

Supplement: Supplemental Information 10 [file peerj-11-16237-s010.zip › Raw data-Ki67-CAL27/cal27 shHtrA3/5blue.tif]

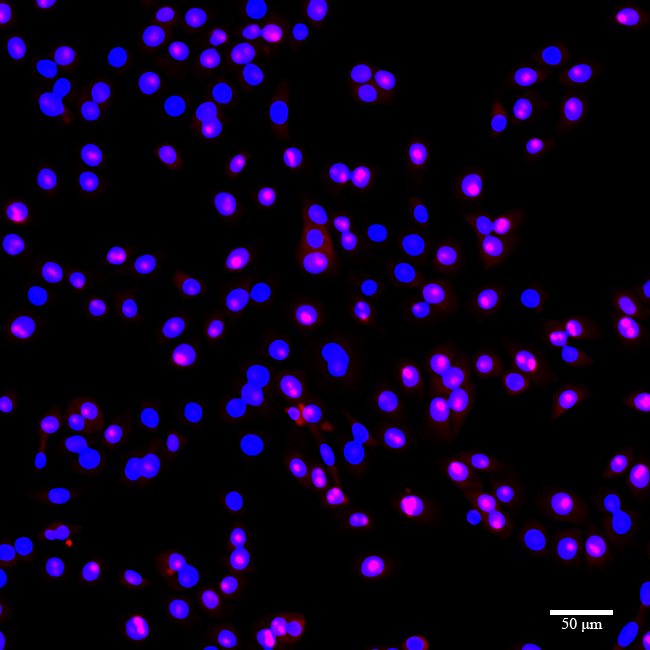

Supplement: Supplemental Information 10 [file peerj-11-16237-s010.zip › Raw data-Ki67-CAL27/cal27 shHtrA3/5merge.tif]

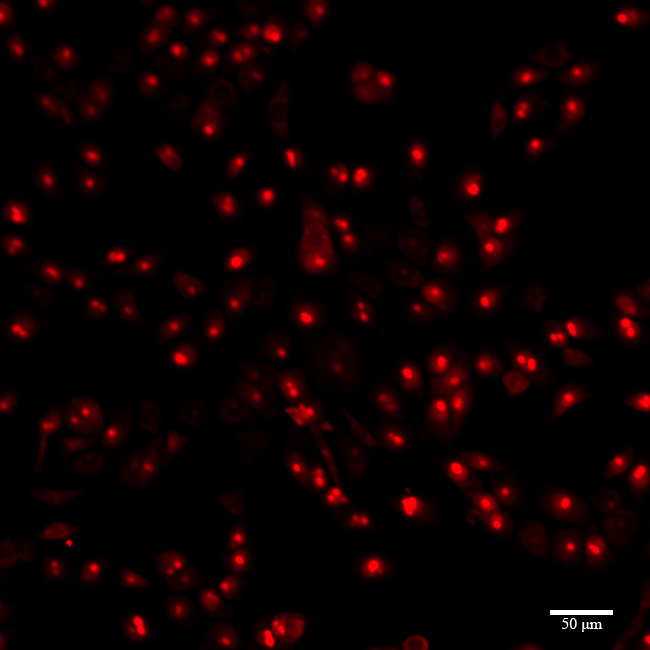

Supplement: Supplemental Information 10 [file peerj-11-16237-s010.zip › Raw data-Ki67-CAL27/cal27 shHtrA3/5red.tif]

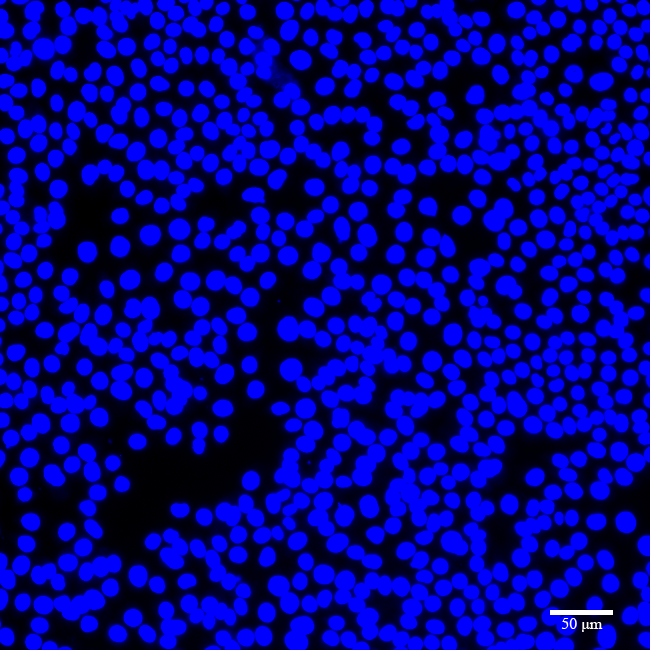

Supplement: Supplemental Information 10 [file peerj-11-16237-s010.zip › Raw data-Ki67-CAL27/cal27 shNC/1blue.tif]

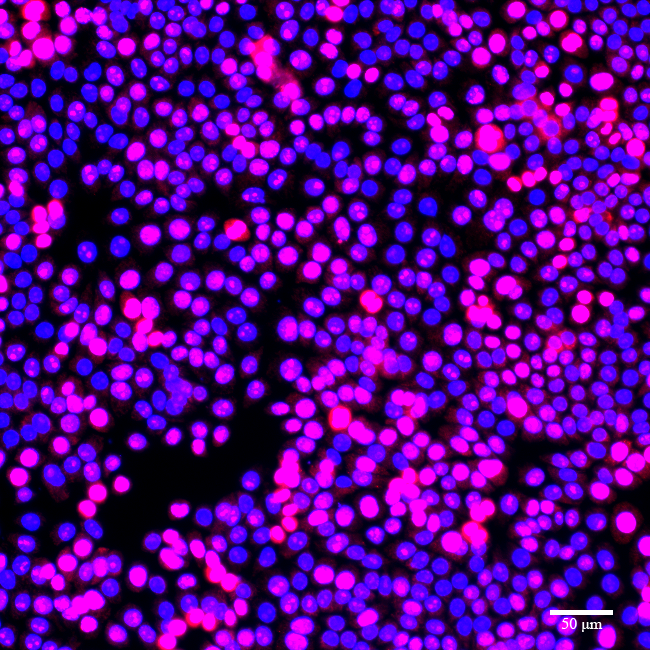

Supplement: Supplemental Information 10 [file peerj-11-16237-s010.zip › Raw data-Ki67-CAL27/cal27 shNC/1merge.tif]

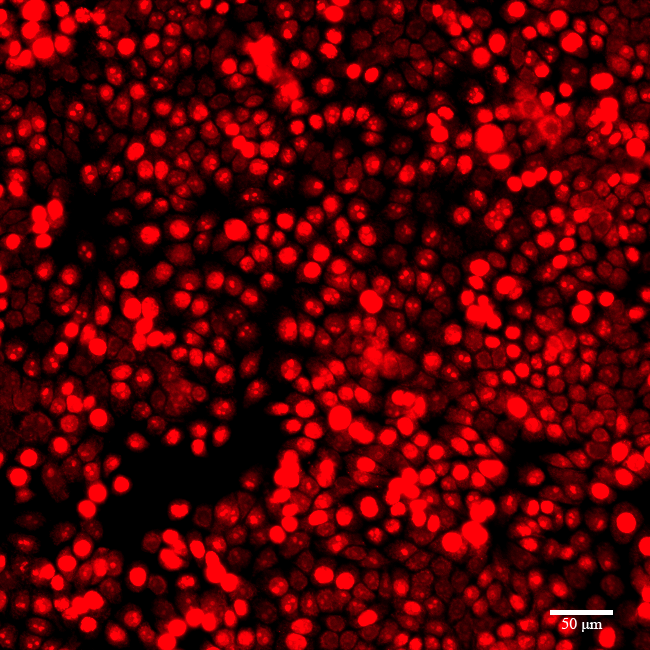

Supplement: Supplemental Information 10 [file peerj-11-16237-s010.zip › Raw data-Ki67-CAL27/cal27 shNC/1red.tif]

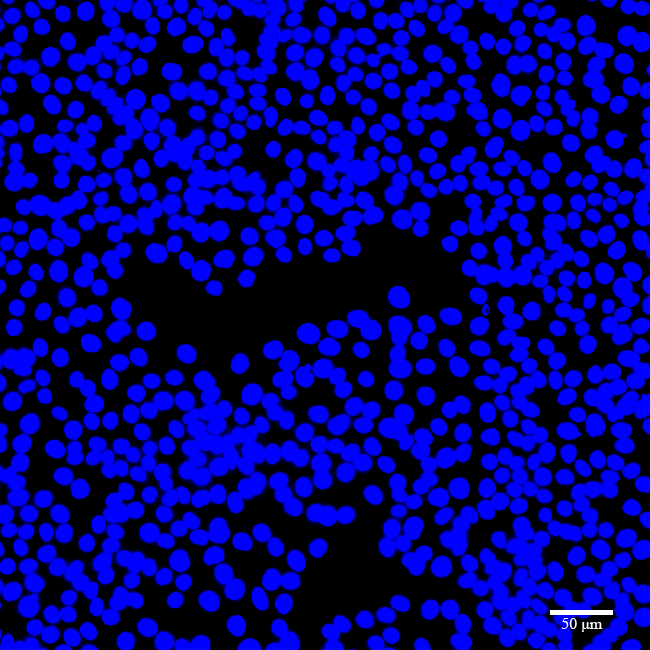

Supplement: Supplemental Information 10 [file peerj-11-16237-s010.zip › Raw data-Ki67-CAL27/cal27 shNC/2blue.tif]

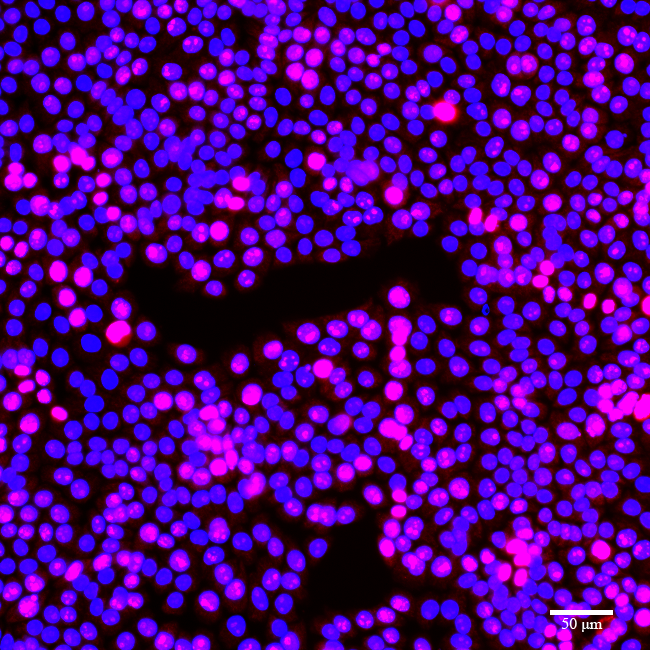

Supplement: Supplemental Information 10 [file peerj-11-16237-s010.zip › Raw data-Ki67-CAL27/cal27 shNC/2merge.tif]

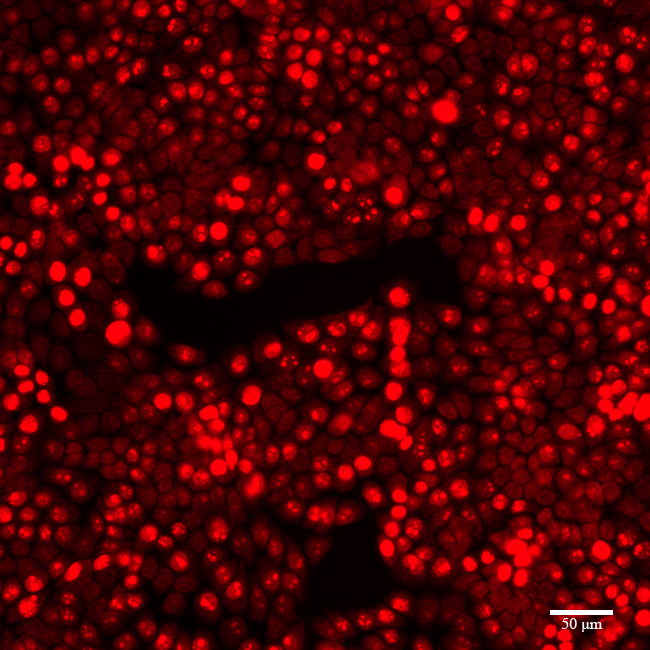

Supplement: Supplemental Information 10 [file peerj-11-16237-s010.zip › Raw data-Ki67-CAL27/cal27 shNC/2red.tif]

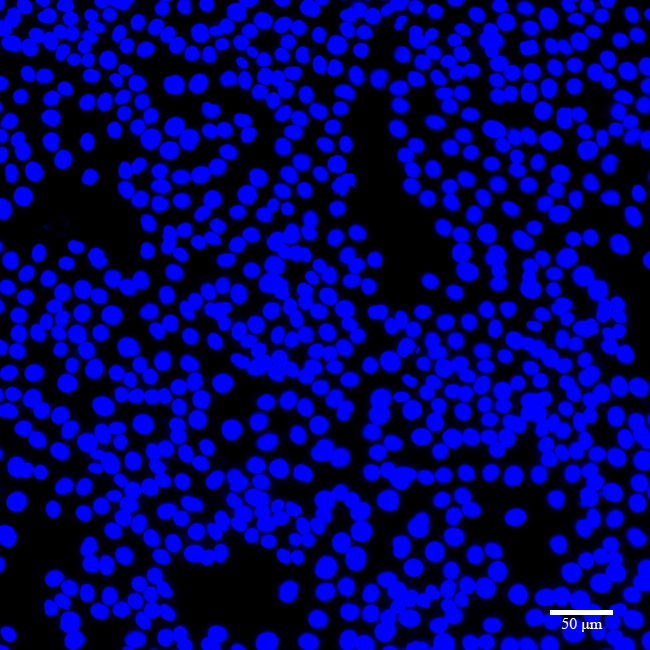

Supplement: Supplemental Information 10 [file peerj-11-16237-s010.zip › Raw data-Ki67-CAL27/cal27 shNC/3blue.tif]

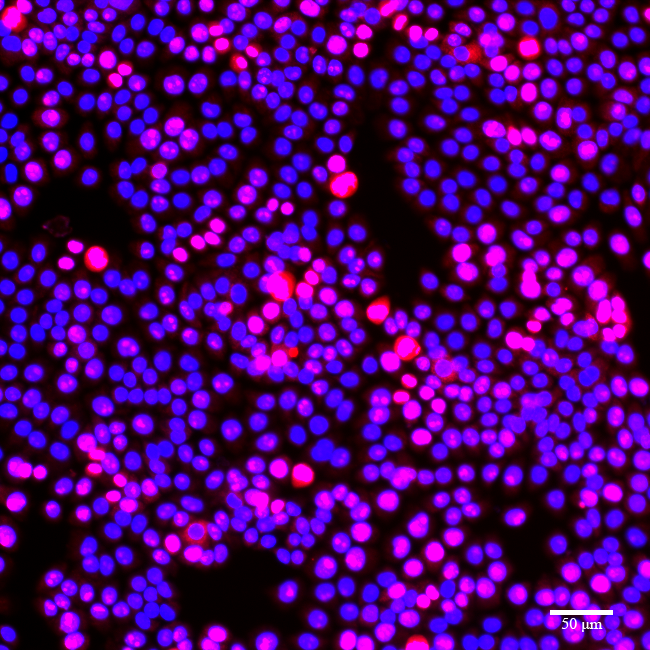

Supplement: Supplemental Information 10 [file peerj-11-16237-s010.zip › Raw data-Ki67-CAL27/cal27 shNC/3merge.tif]

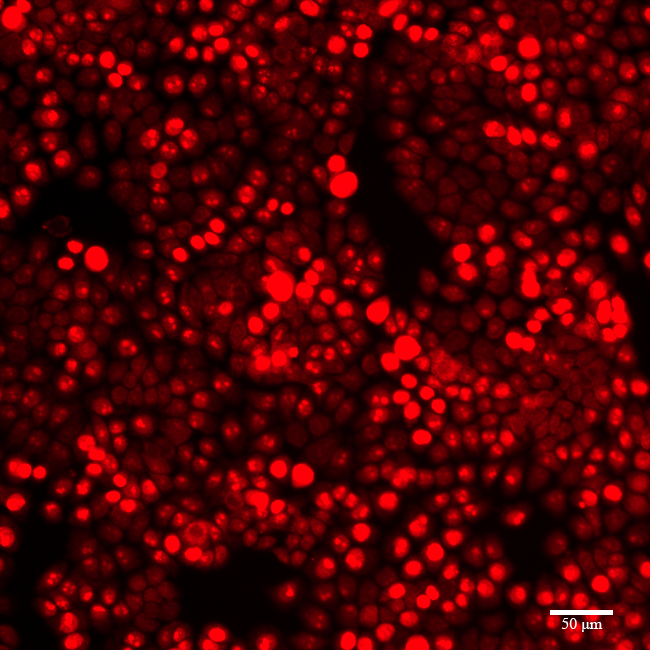

Supplement: Supplemental Information 10 [file peerj-11-16237-s010.zip › Raw data-Ki67-CAL27/cal27 shNC/3red.tif]

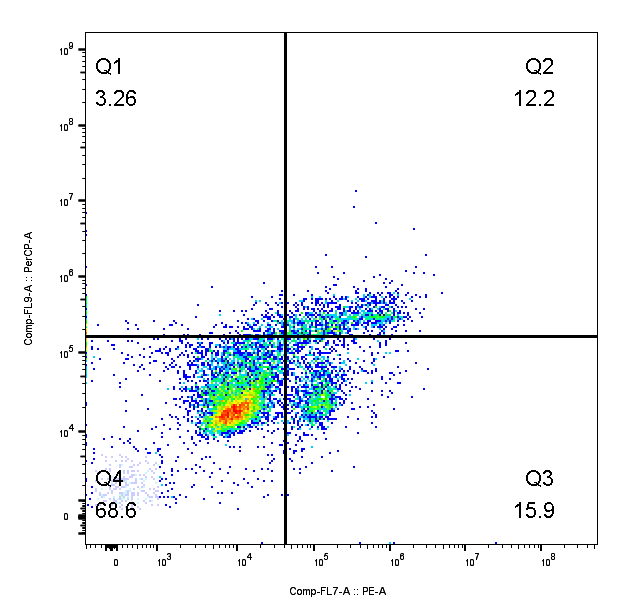

Supplement: Supplemental Information 11 [file peerj-11-16237-s011.zip › Raw data-Apoptosis/CAL-27 shHtrA3 4.fcs.png]

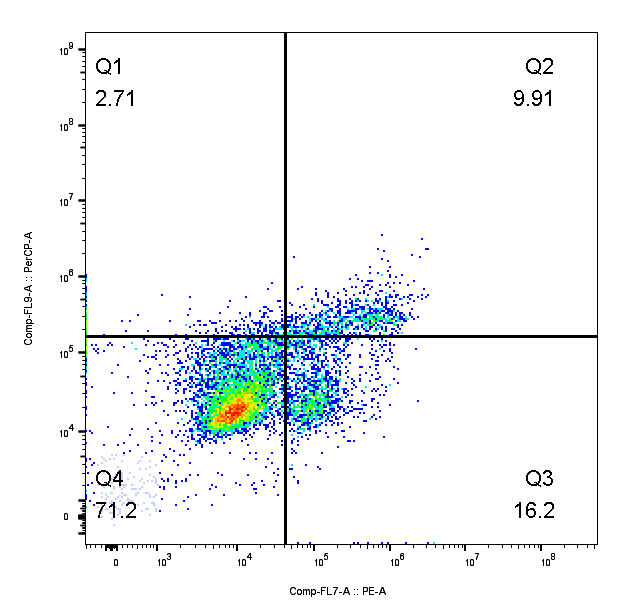

Supplement: Supplemental Information 11 [file peerj-11-16237-s011.zip › Raw data-Apoptosis/CAL-27 shHtrA3 6.fcs.png]

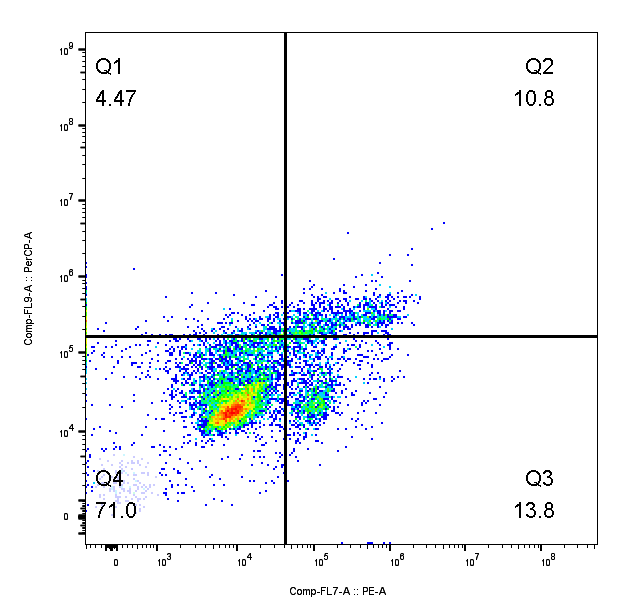

Supplement: Supplemental Information 11 [file peerj-11-16237-s011.zip › Raw data-Apoptosis/CAL-27 shHtrA3 7.fcs.png]

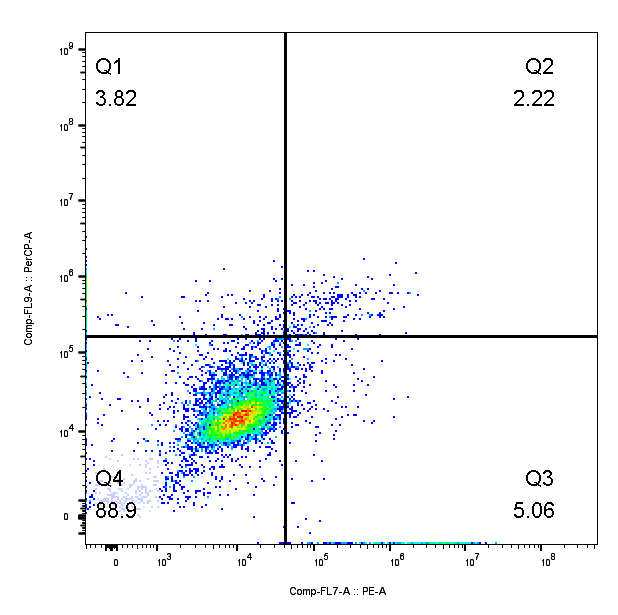

Supplement: Supplemental Information 11 [file peerj-11-16237-s011.zip › Raw data-Apoptosis/CAL-27 shNC 10.fcs.png]

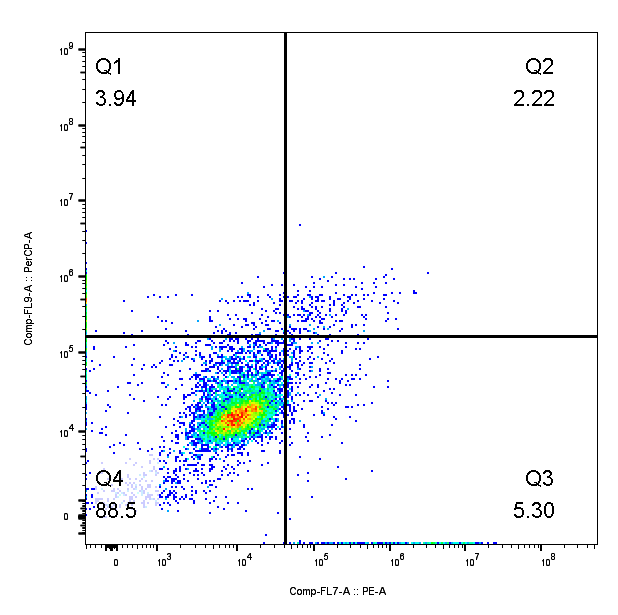

Supplement: Supplemental Information 11 [file peerj-11-16237-s011.zip › Raw data-Apoptosis/CAL-27 shNC 15.fcs.png]

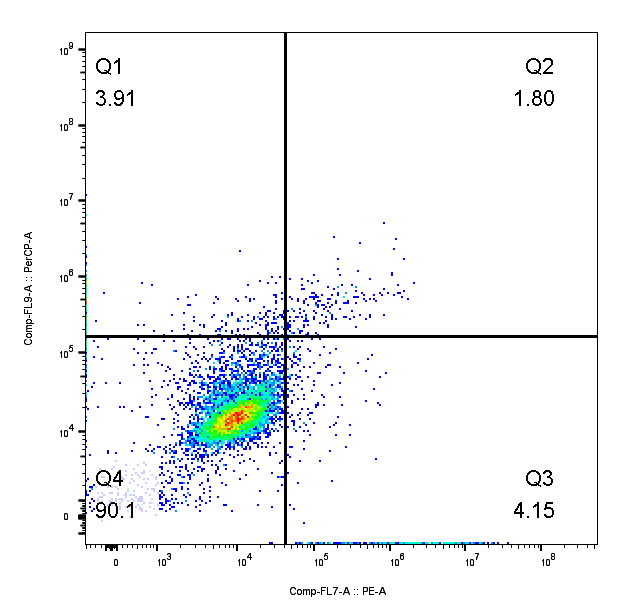

Supplement: Supplemental Information 11 [file peerj-11-16237-s011.zip › Raw data-Apoptosis/CAL-27 shNC 18.fcs.png]

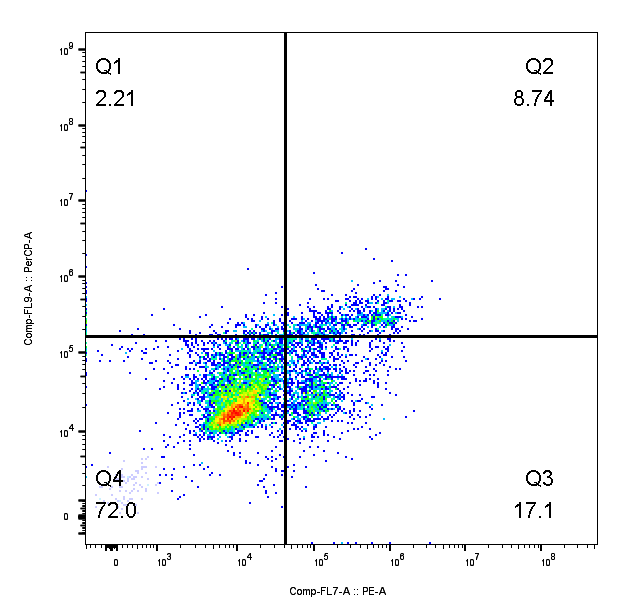

Supplement: Supplemental Information 11 [file peerj-11-16237-s011.zip › Raw data-Apoptosis/fadu shHtrA3 1.fcs.png]

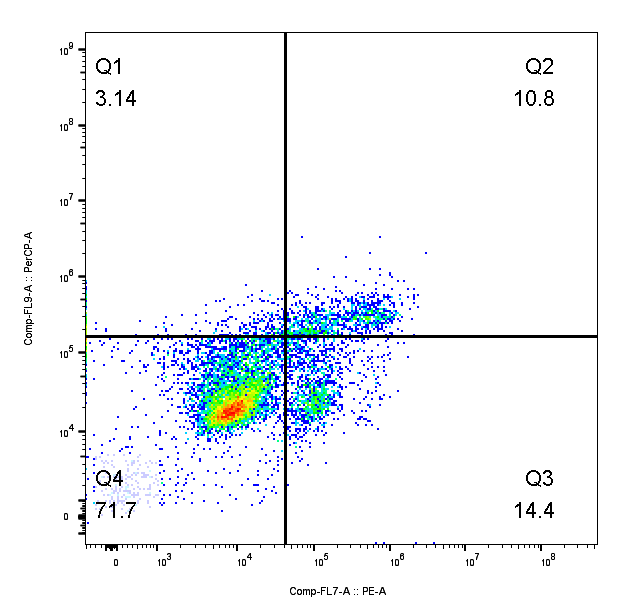

Supplement: Supplemental Information 11 [file peerj-11-16237-s011.zip › Raw data-Apoptosis/fadu shHtrA3 2.fcs.png]

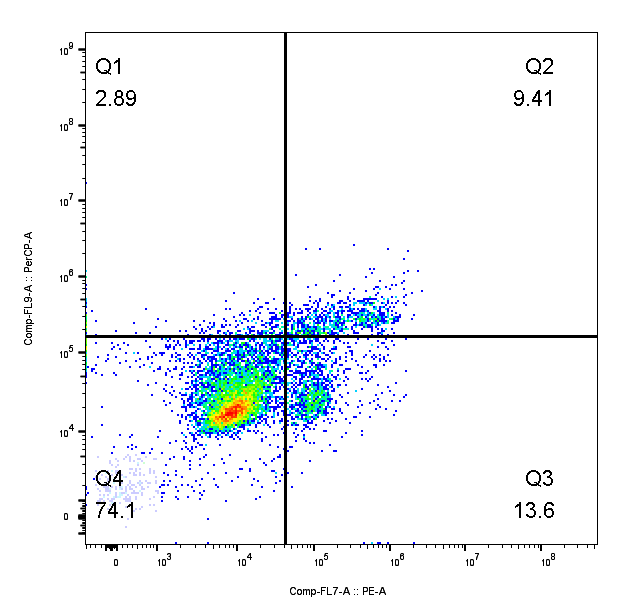

Supplement: Supplemental Information 11 [file peerj-11-16237-s011.zip › Raw data-Apoptosis/fadu shHtrA3 3.fcs.png]

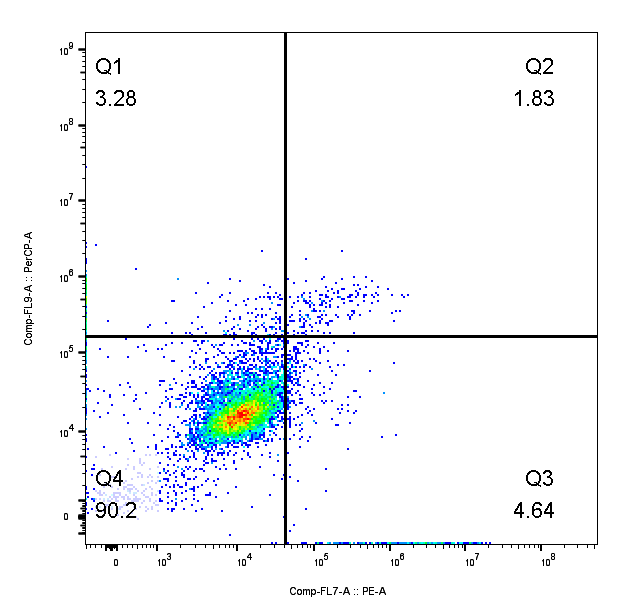

Supplement: Supplemental Information 11 [file peerj-11-16237-s011.zip › Raw data-Apoptosis/fadu shNC 13.fcs.png]

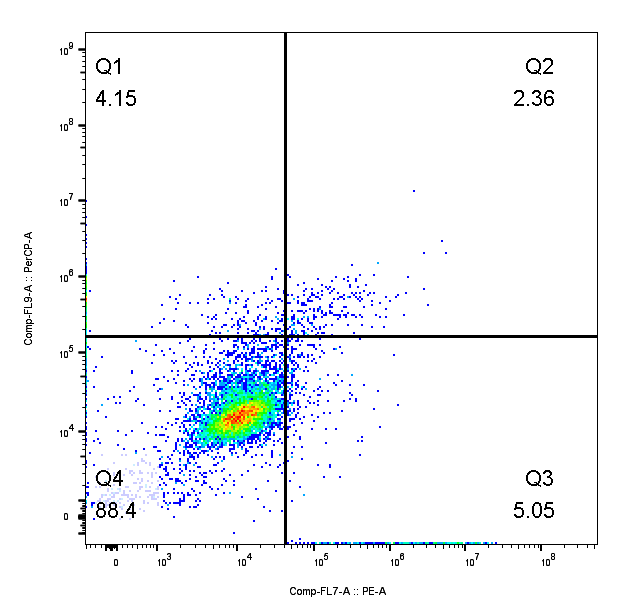

Supplement: Supplemental Information 11 [file peerj-11-16237-s011.zip › Raw data-Apoptosis/fadu shNC 16.fcs.png]

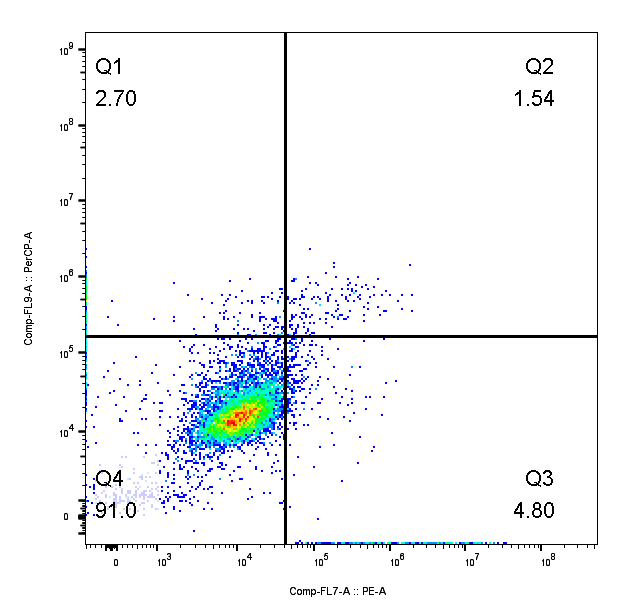

Supplement: Supplemental Information 11 [file peerj-11-16237-s011.zip › Raw data-Apoptosis/fadu shNC 8.fcs.png]
